# Nitrite Reductases in Biomedicine: From Natural Enzymes to Artificial Mimics

**DOI:** 10.34133/research.0710

**Published:** 2025-05-28

**Authors:** Sai Zhu, Zhengbiao Liu, Bo Hu, Yonghai Feng, Guoqing Pan

**Affiliations:** ^1^ Institute for Advanced Materials, School of Materials Science and Engineering, Jiangsu University, Zhenjiang 212013, China.; ^2^ Department of Orthopedics, Suzhou Industrial Park Xinghu Hospital, Suzhou, Jiangsu 215000, China.; ^3^ Jilin Provincial Key Laboratory of Western Jilin’s Clean Energy, Baicheng Normal University, Baicheng 137000, China.

## Abstract

Nitrite reductases (NiRs) are natural enzymes that facilitate the reduction of nitrite. They are essential for the microbial nitrogen cycle and play a vital role in regulating numerous physiological and pathological processes associated with nitric oxide (NO) in living organisms. By the merits of protein engineering, a variety of artificial NiR mimics have been developed. These include traditional artificial proteins, metal-azacycle complexes, and nanozymes such as metal, metal oxide/sulfide nanoparticles, metal-organic frameworks, bioinorganic nanohybrids, and advanced single-atom nanozymes. This development marks an important milestone in broadening the application of enzyme-like catalytic nitrite reduction across various fields, such as biomedicine, biosensing, food science, and environmental science. In this review, we first outline the different types of NiRs, along with their active center structures and catalytic mechanisms, drawing from recent research and discoveries. We then classify the reported NiR mimic materials, discussing their active center structures and enzyme-like catalytic mechanisms. Additionally, we explore the potential future applications and challenges facing NiR mimics in the field of biomedicine.

## Introduction

**Fig. 1.**
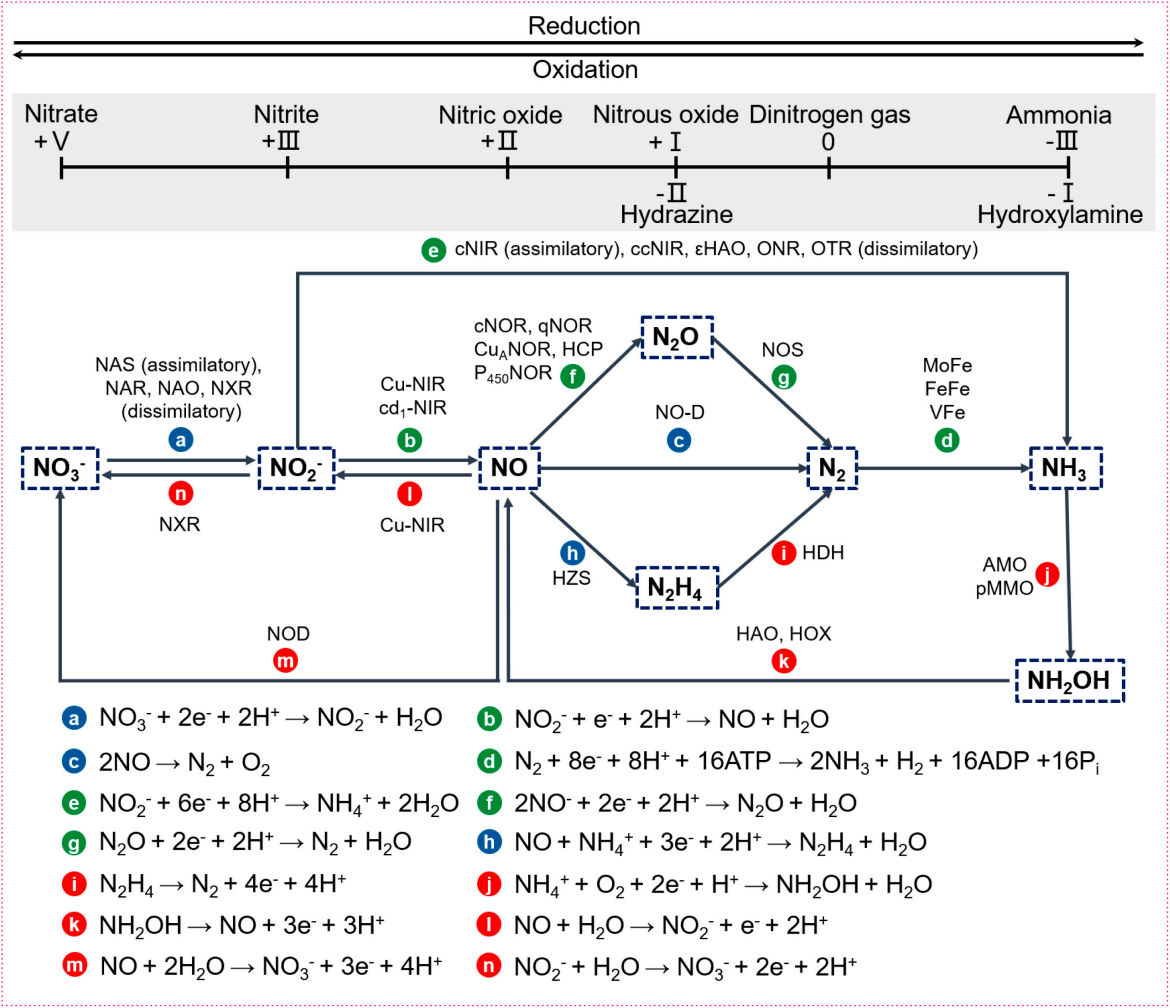
Schematic illustration of the role of nitrite reduction (route b and route e) catalyzed by NiRs in the nitrogen cycle.

The reduction of nitrite (NO_2_^–^) is crucial in nitrogen cycles, particularly in microbial processes, and also serves as a key pathway for maintaining nitric oxide (NO) balance in mammals. Nitrite reductases (NiRs) play an essential role in this process and are found across various microorganisms, plants, and animals. Based on their products, NiRs can be classified into 2 main categories: one group catalyzes the reduction of nitrite to NO, including iron-containing enzymes such as cytochrome cd1 nitrite reductase (cd1NiR) and copper-containing enzymes like CuNiRs [1]. The other group reduces nitrite to ammonia (NH_3_) or ammonium (NH_4_^+^), exemplified by cytochrome c nitrite reductase (ccNiR) and assimilatory nitrite reductase (aNiR) [2]. In microorganisms, NiRs typically operate through a one-electron transfer process represented by the reaction NO_2_^−^ + e^−^ + 2H^+^ → NO + H_2_O (route b, Fig. 1), producing NO [3]. Alternatively, a 6-electron reduction process occurs as NO_2_^−^ + 6e^−^ + 8H^+^ → NH_4_^+^+ 2H_2_O (route e, Fig. 1), leading to the formation of ammonia [4]. These reactions are critical in the biological nitrogen cycle and contribute to anaerobic energy metabolism during dissimilatory nitrate ammonification (Fig. 1) [5]. In humans and animals, NiRs such as heme- and molybdopterin-based proteins are vital for mitigating NO deficiency resulting from reduced nitric oxide synthase (NOS) activity under hypoxic conditions [6]. For instance, hemoglobin regulates blood pressure (BP), hypoxic vasodilation, platelet activation, and cellular adaptation to low oxygen levels through the reduction of nitrite to NO [7,8].

As a highly reactive diatomic free radical, NO influences cellular functions and shows potential in antibacterial and anticancer therapies [9,10]. Moreover, the synthesis of ammonia (NH_3_) is critical for modern industry and agriculture, further highlighting the importance of NiRs [11]. However, the intrinsic instability, low yield, and high production costs of current industrial NiRs remarkably limit their applications. This limitation creates an urgent need for the development of alternative artificial enzymes that exhibit NiR activity. Recent advancements in understanding NiR structures and catalytic mechanisms, along with progress in organic synthesis and nanotechnology, have led to the emergence of a range of effective NiR mimics. These include traditional artificial proteins [12,13], copper–nitrogen complexes [14], iron–nitrogen complexes [15], metal–biomolecule complexes [16], metal nanoparticles (NPs) [17], metal-organic frameworks (MOFs) [18], and advanced single-atom catalysts [19]. Despite the growing interest in novel NiR mimics, especially for biomedical applications, comprehensive reviews focusing on advancements in artificial NiR mimics are lacking. This review aims to fill that gap by summarizing recent developments in NiRs and their artificial counterparts. It categorizes NiRs and their corresponding mimics, compares and discusses their catalytic mechanisms, highlights the applications of NiR mimics in biomedicine, and explores future prospects and challenges in the field.

## Natural NiRs

### Classification of NiRs

As mentioned above, NiRs can be categorized based on their products into 2 main types: NO-producing and ammonia-producing NiRs. They can also be classified by the metal type—copper, iron, or molybdenum—and by their origin from bacteria or animals. Accordingly, we classify several key NiRs according to their product type, metal active center, and source. As listed in (Table 1), NO-producing NiRs primarily catalyze the reduction of nitrite to NO via a one-electron nitrosylation pathway. This group includes CuNiRs, cd1NiRs, hemoproteins, mitochondrial proteins, molybdenum enzymes, di-iron YtfE proteins, and endothelial NOS (eNOS). Conversely, ammonia-producing NiRs reduce nitrite to ammonia through the transfer of 6 electrons. This category primarily encompasses aNiRs, which are ferredoxin protein-dependent and NAD(P)H-dependent, as well as multiheme ccNiR. Notably, the CuNiRs, cd1NiR, ferredoxin-dependent NiRs, and ccNiRs are key NiRs found in bacteria, while hemoproteins, mitochondrial proteins, molybdenum enzymes, and eNOS are primarily involved in nitrite reduction in animals. NiRs are typical metalloenzymes, with their active sites predominantly composed of metal atoms such as copper, iron, or molybdenum, each situated within a specific coordination environment[7–10].

**Table 1.** Active sites, structures, activities and functions of typical NiRs

Nitrite reductases		Nitrogen cycle	Product	Active centers	Structures	Activity			Functions	Ref.
						*K*_m_ (μM)	*V*_max_ (μmol/min/mg)	Rate constant (nM/s)		
Copper containing nitrite reductases (CuNiR) (*Alcaligenes xylosoxidans*)		Denitrification	NO	Cu	A homotrimeric protein comprises 3 identical subunits, each consisting of 2 distinct domains.	250	570	-	It catalyzes the reduction of nitrite to form nitric oxide (NO), a crucial step in the nitrogen cycle.	[136,137]
Cytochrome cd1 (cd1NiR) (*Paracoccus pantotrophus*)		Denitrification	NO	Fe	A homodimer consists of 2 identical subunits, each comprising a heme c-containing domain and a heme d1-containing domain.	170	0.6	-	It catalyzes the reduction of nitrite to NO, which is a key step in the denitrification pathway of the biological nitrogen cycle.	[3,138]
Hemeproteins	Hemoglobin (Hb)	Denitrification	NO	Fe	It contains 2 α and 2 β subunits, each bound to a heme moiety.	-	-	12	It is responsible for the transport and delivery of oxygen to tissues and organs in animals.	[139]
	Myoglobin (Mb)				A globular protein composed of 153 residues, this protein binds molecular O_2_ and other small ligands at a ferrous (Fe^2+^) heme iron.	-	-	10	It primarily facilitates the delivery of oxygen to mitochondria in red muscle, promotes O_2_ diffusion, and aids in NO detoxification.	[17,140]
	Neuroglobin (Ngb)				A globin alignment in the retina, characterized by conserved α-helices and structural motifs typical of the classical globin fold.	-	-	0.5	It plays a key role in mitigating stroke-related damage in the brain and protecting the retina, an area of high O_2_ consumption, from O_2_ deprivation.	[18]
	Cytoglobin (Cyb)				It is dimerized through 2 intermolecular disulfide bonds between Cys38 (B2) and Cys83 (E9).	-	-	0.35	It facilitates the transfer of oxygen from arterial blood to the brain.	[141]
	Alpha globin				A histidine side chain coordinates the ferrous iron atom in the center of the bound heme.	-	-	35	Its structural instability as a monomer renders it susceptible to oxidation and precipitation, likely contributing to β-thalassemia and other blood disorders.	[14,15,142]
Mitochondrial proteins	Complex III (cytochrome bc1 complex)			Fe, Fe_2_S_2_ cluster	An integral part of the mitochondrial respiratory chain	-	-		Plays a central role in cellular energy production	[24,143]
	Cytochrome c			Fe	A heme protein located in the intermembrane space between the inner and outer mitochondrial membranes binds one or more c-type hemes via 2 thioether bonds formed by the sulfhydryl groups of 2 cysteine residues.	-	-	14	It is essential for cell survival, as it serves as an electron carrier between Complex III and Complex IV of the electron transport chain, thereby facilitating ATP generation.	[144,145]
	Cytochrome c oxidase (CcOx)			Cu, Fe	The redox-active metal sites, designated as CuA, heme a, CuB, and heme a3, present 2 hydrophilic surfaces that face the P-phase and the N-phase, respectively.	-	-	1.5	As the terminal oxidase of cellular respiration, this complex reduces molecular O_2_ in a reaction that is coupled with proton pumping.	[146]
Molybdenum metalloenzymes	Xanthine oxidoreductase (XOR)			Mo	This enzyme contains one molybdopterin (Mo-pt) cofactor, 2 distinct [2Fe–2S] clusters, and one FAD cofactor.	-	-	1	It functions as a highly effective nitrite/nitrate reductase, playing a crucial role in catalyzing NO generation from nitrite in mammalian tissues, particularly under acidic conditions.	[147]
	Aldehyde oxidase (AO)				These enzymes contain 2 iron–sulfur clusters, a flavin cofactor, and a molybdopterin cofactor.	-	-	1.5	They catalyze reactions involving the transfer of an oxygen atom to or from the substrate, playing a marked role in the global carbon, nitrogen, and sulfur cycles.	[44]
	Sulfite oxidase (SO)				Each subunit comprises 3 distinct domains: a molybdenum cofactor domain, a dimerization domain, and a smaller heme-containing domain.	-	-	-	This enzyme oxidizes sulfite to sulfate and transfers the electrons produced to the electron transport chain via cytochrome c, facilitating ATP generation during oxidative phosphorylation.	[48]
	Mitochondrial amidoxime reductase component (mARC)				mARC proteins exhibit marked sequence similarity to the C-terminal domains of eukaryotic molybdenum cofactor sulfurases.	-	-	-	They play an important role in nitrogen-reductive activity and energy metabolism.	[148,149]
Di-iron protein YtfE				di-Fe	This monomeric L-shaped molecule consists of 2 domains.	88	0.04	-	It contributes to protection against nitrosative stress and enhances survival within host tissues.	[27]
Endothelial nitric oxide synthase (eNOS)				Fe	This protein consists of a heme domain connected via a calmodulin-binding linker peptide to an NADPH-cytochrome P450 reductase-like diflavin domain.	-	-	0.18	It is a constitutively expressed enzyme that oxidizes L-arginine to generate L-citrulline and NO.	[150,151]
Assimilatory nitrite reductases (aNiRs)	fdNiRs	Reduction	NH_4_^+^	Fe	An HS ferric siroheme is a complex heme derivative comprising an iron-containing isobacteriochlorin extensively substituted with carboxylic acid side chains and bridged to a tetranuclear [4Fe–4S] cluster via a common cysteine ligand.	100	26	-	This enzyme catalyzes the 6-electron reduction of nitrite to ammonia.	[12,13,152,153]
	NAD(P)H-NiRs					5	0.3	-		
ccNiRs	*Desulfovibrio desulfuricans*	Reduction	NH_4_^+^	Fe	This enzyme functions as a dimer, featuring 10 closely packed c-type heme groups and an unusual lysine-coordinated high-spin heme at the active site.	1,140	453	-	It catalyzes the 6-electron reduction of nitrite to ammonia, a key step in the biological nitrogen cycle and anaerobic energy metabolism of dissimilatory nitrate ammonification.	[4,51]
	*Escherichia coli*					870	28	-		

### Structures and active centers of NiRs

#### Cu-containing NiRs

**Fig. 2.**
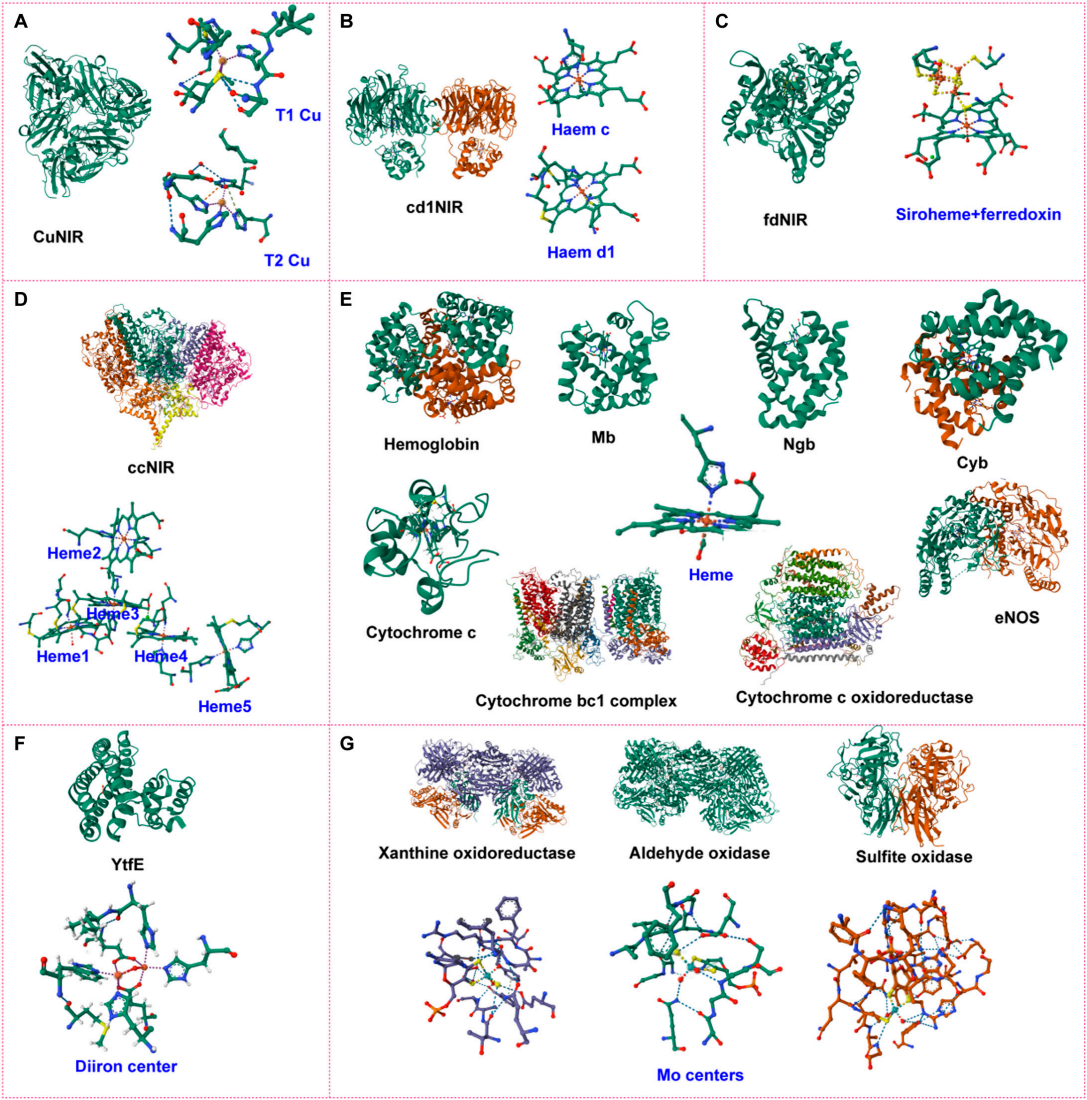
Structures and active metal centers of NiRs. (A) CuNiR; (B) cd1NiR; (C) fdNiR; (D) ccNiR; (E) hemoglobin, myoglobin (Mb), neuroglobin (Ngb), cytoglobin (Cyb), cytochrome c, cytochrome bc1 complex, cytochrome c oxidoreductase, and endothelial nitric oxide synthase (eNOS); (F) YtfE; and (G) xanthine oxidoreductase, aldehyde oxidase, and sulfite oxidase.

Cu-containing NiRs (CuNiRs) typically function as trimers, with each enzyme molecule comprising 3 identical subunits (Fig. 2A). These subunits interact via noncovalent bonds to form a stable trimer. Each subunit contains 2 copper ions: Type 1 Copper (T1 Cu) and Type 2 Copper (T2 Cu). T1 Cu plays a critical role in electron transfer, typically coordinated by cysteine, histidine, and methionine residues, while T2 Cu functions as the active center for nitrite reduction. Specifically, T2 Cu directly participates in the reduction process by coordinating with 3 histidine residues and a solvent molecule [11]. This catalytic site binds the nitrite molecule to the copper ion, facilitating its reduction to NO [1]. Electrons are transferred from an electron donor, such as pseudoazurin or cytochrome c, through T1 Cu to T2 Cu, thereby driving the nitrite reduction reaction.

#### Fe-containing NiRs

##### Cytochrome cd1NiR

cd1NiR is a dimer composed of 2 identical subunits, each with a molecular weight of 60 kDa [3]. Each subunit consists of 2 domains: the c-type domain and the d1-type domain (Fig. 2B). The c-type domain features an α-helical structure where the haem c group is bound to the protein at the top via 2 thioether bonds. Its primary function is to accept electrons from external donors and transfer them to the catalytic site. The d1-type domain has a unique β-propeller structure and contains the haem d1, which is specific to cytochrome cd1. The haem d1 binding region is located at the bottom of the protein and serves as the active site for nitrite reduction. Haem d1 possesses a cyclic tetrapyrrole structure that differs from the standard tetrapyrrole structure of haem c. The iron atom at the center of haem d1 is the core of the redox reaction. Surrounding the iron atoms in both haem c and haem d1 are various coordinating ligands, primarily histidine residues from the protein backbone, which enhance the stability of the iron centers. Additionally, other amino acid residues may also coordinate with the iron atoms, potentially providing additional electron donor or acceptor functions.

##### Ferredoxin-dependent nitrite reductase

Ferredoxin-dependent nitrite reductase (fdNiR) is a globular protein consisting of 3 structural domains, featuring a [4Fe–4S] cluster and a siroheme cofactor situated between these domains (Fig. 2C) [12]. Siroheme, which functions similarly to heme, catalyzes the 6-electron reduction of nitrite to ammonia [13]. It is linked to the [4Fe–4S]^2+^ cluster via a bridging sulfur atom from a cysteine residue. This [4Fe–4S] cluster is associated with ferredoxin, a small iron–sulfur protein containing a [2Fe–2S] cluster that acts as an electron “capacitor” enabling the oxidation state of the iron atoms to oscillate between +2 and +3. The electron flow proceeds from the reduced ferredoxin to the [4Fe–4S] cluster of fdNiR, followed by transfer from the reduced cluster to the siroheme, ultimately facilitating the reduction of nitrite ions to ammonia.

##### Cytochrome c nitrite reductase

ccNiR is a homodimer, with each protomer having a mass ranging from 52 to 65 kDa, depending on the organism of origin. Each protomer contains 5 c-type hemes, all in the iron (III) state in the resting enzyme (Fig. 2D) [4]. The active site (heme 1) is high-spin, featuring a lysine residue in the proximal position and a labile water molecule in the distal position. The other 4 hemes (hemes 2 to 5) are hexacoordinated, bound by 2 histidine residues, and exist in a low-spin state. Electrons are believed to enter the enzyme through heme 2, transferring sequentially between hemes until they reach the active site of either protomer. In the heme c molecule, the iron atom is centrally located and coordinated by the nitrogen atoms of the 4 pyrrole rings. Additionally, the iron atom can also be coordinated with histidine residues and other ligands within the protein.

##### Heme-based nitrite reductases (hemeproteins)

Hemoglobin is primarily recognized for its role in oxygen transport within red blood cells, but it also catalyzes the reduction of nitrite ions to produce the vasoreactive molecule NO, which serves as an important source of NO under hypoxic conditions [14]. This function is largely attributed to the heme group in hemoglobin, which consists of a porphyrin ring with a central iron atom coordinated by 4 nitrogen atoms of the ring [15]. Under hypoxic conditions, the deoxygenated heme group interacts with nitrite, facilitating its reduction to NO. Following this reaction, the iron in the heme is reduced back from the ferric state (Fe^3+^) to the ferrous state (Fe^+^), allowing it to either bind another gas molecule or react with nitrite again [16]. In animals, several hemeproteins, including hemoglobin A (Hb), myoglobin (Mb), neuroglobin (Ngb), cytoglobin (Cyb), the hemoglobin α subunit, mitochondrial membrane proteins such as the cytochrome bc1 complex, cytochrome c, cytochrome c oxidase, and eNOS, exhibit NiR activity under hypoxic conditions [6]. This activity is primarily due to structural similarities in their active centers (Fig. 2E):

1. *Heme group*: Hemoglobin [15], Mb [17], Ngb [18], Cyb [19], cytochrome c [4], and eNOS [20] all contain a heme group featuring a porphyrin ring with a central iron atom. Cytochrome c oxidase possesses a binuclear center composed of heme a and heme a3-Cu_B [21].
2. *Iron atom coordination*: In hemoglobin, Mb, Ngb, Cygb, cytochrome c, and eNOS, the iron atom is coordinated by the 4 nitrogen atoms of the porphyrin ring and a histidine residue (Fig. 2). In the cytochrome bc1 complex, the iron in cytochrome b is coordinated by the nitrogen atoms of the porphyrin ring, while the Rieske iron–sulfur center contains a [2Fe–2S] cluster [22,23].
3. *Electron transfer function*: The cytochrome bc1 complex, cytochrome c, and cytochrome c oxidase are critical components of the mitochondrial electron transport chain, responsible for efficient electron transfer [24]. eNOS generates NO through its active center, which involves oxygen and electron transfer [20]. Conversely, hemoglobin, Mb, Ngb, and Cygb are primarily engaged in oxygen binding and transport, enabling the reversible binding and release of oxygen [15].
4. *Cofactors*: In eNOS, tetrahydrobiopterin (BH4) acts as a cofactor [25], similar to the copper ions in cytochrome c oxidase [21] and the iron–sulfur clusters in the cytochrome bc1 complex [26]. These cofactors help stabilize the enzyme’s active conformation and promote the reaction.

##### Di-iron YtfE proteins

The YtfE enzyme is a disulfide bond reductase found in bacteria and is classified within the non-heme diiron oxidoreductase family [27]. Its protein structure typically features multiple α-helices and β-sheets. The catalytic function of YtfE centers around its diiron center, which is crucial for its activity (Fig. 2F). The coordination environment of this diiron center plays a vital role, with amino acid residues such as histidine, glutamate, and aspartate coordinating with the iron atoms through their nitrogen or oxygen atoms. This stable coordination is further enhanced by bridging ligands, such as hydroxide (OH^−^) or water molecules (H_2_O), which connect the 2 iron atoms. Collectively, these amino acid residues and bridging ligands create a highly specialized environment that facilitates efficient electron transfer and catalytic activity at the diiron center.

#### Molybdenum metalloenzymes

In addition to copper- and iron-based NiRs, enzymes with molybdenum (Mo) as the active center, such as xanthine oxidoreductase [28,29], aldehyde oxidase [30], sulfite oxidase [31], and mitochondrial amidoxime reductase component [32], also demonstrate high NiR activity under hypoxic conditions. The active centers of these enzymes contain a molybdenum cofactor, with the molybdenum atom centrally located and coordinated by thiol groups (usually from molybdopterin) and other ligands, including cysteine, histidine, or sulfur/oxygen-containing residues (Fig. 2G). These residues interact closely with the molybdenum cofactor or iron–sulfur clusters, ensuring the stability and functionality of the active center. In all these enzymes, the molybdenum atom forms a multidentate structure, typically exhibiting a hexacoordinated octahedral geometry. This arrangement enables the molybdenum atom to efficiently participate in redox reactions, particularly during nitrite reduction catalysis. Additionally, these enzymes often contain iron–sulfur clusters, such as [2Fe–2S], which play a crucial role in electron transfer by shuttling electrons between flavin adenine dinucleotide (FAD) and the molybdenum cofactor. Although the specific functions of these enzymes may vary, they typically possess an FAD-binding domain responsible for transferring electrons from substrates or other electron donors to the molybdenum cofactor, thereby facilitating the redox reactions.

### Catalytic mechanism of natural NiRs

#### Reduction of nitrite to NO over Cu-, Fe-, and Mo-based NiRs

**Fig. 3.**
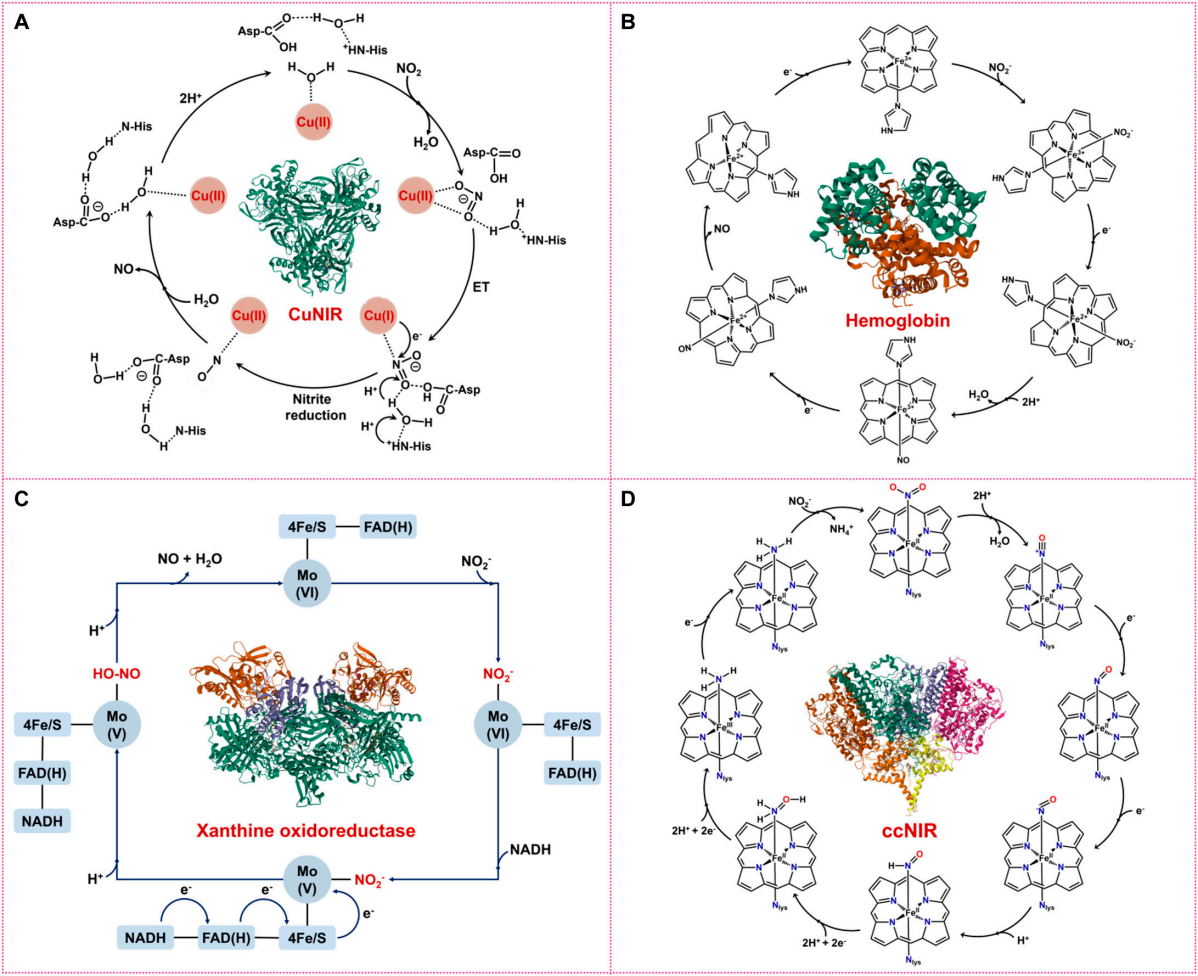
Mechanisms of nitrite reduction catalyzed by NiRs with different active sites. (A) Nitrite reduction to NO over CuNiR, (B) nitrite reduction to NO over heme-based NiRs, (C) nitrite reduction to NO over Mo-based NiRs, and (D) nitrite reduction to NH_3_ over ccNiR.

1. CuNiRs are essential enzymes in the denitrification process, catalyzing the reduction of nitrite to NO. Recent studies have elucidated their catalytic mechanisms, revealing intricate details of proton and electron transfers. As illustrated in Fig. 3A, the reduction of nitrite involves several key steps: First, nitrite binds to the T2 copper center, initially coordinating through one oxygen atom [1]. Then, protons are transferred to the nitrite via a network of water molecules and residues, including Asp98 and His255 [1], which are crucial for proton delivery and maintaining the active site structure [33]. Subsequently, electrons are transferred from the T1 copper site to the T2 site, reducing the nitrite to a nitrosyl intermediate. Finally, the nitrosyl intermediate undergoes further protonation and electron transfer, leading to the release of NO and regeneration of the enzyme’s resting state [34]. Studies have proposed that CuNiRs operate via a random-sequential mechanism, where electron transfer from T1 to T2 is rate-limiting. The order of nitrite binding and reduction can vary depending on substrate concentration and pH, influencing the enzyme’s efficiency [35]. Density functional theory (DFT) calculations have provided detailed insights into the reaction pathways, activation energies, and transition states involved in nitrite reduction. These studies have clarified the roles of protons and electrons in the catalytic cycle and resolved previous controversies regarding the coordination modes of intermediates [1]. In summary, CuNiRs employ a complex mechanism involving coordinated proton and electron transfers to reduce nitrite to NO efficiently. Understanding these processes is crucial for applications in environmental nitrogen cycling and bioinspired catalysis.
2. Heme-based NiRs, including Hb, Mb, Ngb, and Cyb, are well-known for their primary function in oxygen transport. However, under hypoxic conditions, they also serve as NiRs, catalyzing the conversion of nitrite to NO [36–38]. This reaction is particularly crucial during tissue hypoxia, as it promotes vasodilation, regulates cellular respiration, and participates in signal transduction pathways [39,40]. In the absence of oxygen, the ferrous (Fe^2+^) center in deoxy-Hb exhibits a lower redox potential, enabling it to reduce nitrite to NO. During this process, as shown in Fig. 3B, nitrite binds to deoxy-Hb, forming a ferrous-nitrosyl-hemoglobin complex, which releases a water molecule to form a ferric-nitrosyl adduct. Subsequently, NO may react with another deoxy-Hb molecule to form a nitrosylated complex or be released directly as NO gas [41]. In some cases, the ferric heme can be reduced back to the ferrous state by cellular reductases or NADH-dependent reductases, allowing the heme protein to continue participating in nitrite reduction. This reaction can be summarized as: Fe^2+^-Hb + NO₂^−^ + H^+^ → Fe^3+^-Hb + NO + OH^−^. The rate of this reaction is modulated by the conformational state of Hb, pH, and oxygen partial pressure. In the T state (deoxygenated form) of Hb, the ferrous center’s redox potential is lower, favoring nitrite reduction. As the reaction progresses, some T state Hb transitions to the R state (oxygenated form), which has a higher redox potential, further enhancing nitrite reduction [39]. Therefore, Hb exhibits maximal NO generation rates at 40% to 60% oxygen saturation (near the *P*_50_ value) [41]. Additionally, acidic environments (low pH) promote the protonation of nitrite, enhancing its binding to Hb and accelerating NO production [42]. This Hb-mediated nitrite reduction not only serves as a mechanism for oxygen and acidity sensing but also plays an important role in hypoxic vasodilation and NO signaling. Moreover, other heme-containing proteins, such as Mb, exhibit similar NiR activities and may regulate cellular responses to hypoxia in specific tissues [36–38]. These findings provide new insights into the multifunctionality of Hb under physiological and pathological conditions.
3. Molybdenum-based NiRs are a class of enzymes that utilize a molybdenum cofactor (MoCo) to catalyze the reduction of nitrite to NO. In mammals, several molybdenum-containing enzymes have been identified to possess NiR activity, including xanthine oxidase (XO), aldehyde oxidase (AO), sulfite oxidase (SO), and mitochondrial amidoxime-reducing component (mARC). These enzymes share a common catalytic mechanism but differ in their structural features and electron transfer pathways. The catalytic cycle begins with the binding of nitrite to the reduced molybdenum center of the enzyme. As shown in Fig. 3C, the Mo(VI) site of AO then interacts with nitrite to form a coordination complex [43]. Electron transfer from a suitable donor, such as nicotinamide adenine dinucleotide (NADH) or aldehydes, reduces the molybdenum from its hexavalent [Mo(VI)] to pentavalent [Mo(V)] state [44]. This reduction facilitates the transfer of electrons to the nitrite, leading to the formation of a nitrosonium ion (NO^+^). Protons (H^+^) are also transferred during this process, resulting in the production of NO and water [45]. The molybdenum center is then reoxidized to its Mo^6+^ state, ready for another catalytic cycle.

While all these enzymes utilize the MoCo for nitrite reduction, they differ in their structural and functional characteristics:

*XO and AO:* Both are homodimeric molybdenum flavoproteins containing 2 iron–sulfur [2Fe–2S] centers, one molybdenum center, and one FAD cofactor per monomer. AO, located in the cytosol, catalyzes the oxidation of aldehydes and the hydroxylation of heterocyclic compounds [46]. In the presence of NADH, AO can reduce nitrite to NO, a process that is inhibited by oxygen. Electron transfer occurs from NADH to the molybdenum center via FAD and iron–sulfur clusters [47].

*SO:* This homodimeric enzyme consists of a C-terminal molybdenum domain and an N-terminal heme domain [48]. It is located in the mitochondrial intermembrane space and catalyzes the oxidation of sulfite to sulfate, the final step in the oxidative degradation of sulfur-containing amino acids.

*mARC:* The latest discovered molybdenum enzyme in mammals, mARC does not contain other cofactors except the MoCo. It coordinates with cytochrome b₅ and cytochrome b₅ reductase to catalyze electron transfer from NADH to amidoxime groups [49]. mARC has been shown to reduce N-hydroxylated prodrugs and nitrite to NO, though its physiological role remains unclear.

The efficiency of nitrite reduction by these enzymes is remarkably influenced by oxygen levels, pH, and electron donors [46,47]. For instance, high oxygen concentrations can inhibit nitrite reduction by competing for electrons. Under hypoxic or anoxic conditions, the enzymes preferentially reduce nitrite to NO. Therefore, understanding the catalytic mechanisms and regulatory factors of molybdenum-based NiRs provide insights into their roles in NO metabolism and their potential therapeutic applications.

#### Reduction of nitrite to ammonia over Fe-based NiRs

The reduction of nitrite to ammonia (NH_3_) by Fe-based ccNiR and fdNiR begins with the heterolytic cleavage of the N–O bond. This process is facilitated by a strong back-bonding interaction between nitrite, coordinated through nitrogen, and the reduced Fe(II) active site iron, but not the oxidized Fe(III) form [50]. Thus, as shown in (Fig. 3D), nitrite first binds to the Fe(II) active site of ccNiR (or fdNiR), typically at the iron atom in the heme group [12]. The nitrite then accepts the first electron, reducing it to NO while 2 protons (H^+^) are added, resulting in the release of a water molecule: NO_2_^−^ + e^−^ + 2H^+^ → NO + H_2_O [4,51]. Next, NO accepts another electron and one proton to form the nitroxyl (HNO) intermediate: NO + e^−^ + H^+^ → HNO [4,51]. The HNO intermediate then accepts 2 more electrons and 2 protons to produce hydroxylamine (NH₂OH): HNO + 2e^−^ + 2H^+^ → NH_2_OH [4,51]. Hydroxylamine is further reduced by accepting 2 additional electrons and 2 protons, yielding ammonia and water: NH_2_OH + 2e^−^ + 2H^+^ → NH_3_ + H_2_O [4,51]. Throughout this process, the iron atom in the heme group alternates between the reduced ferrous state (Fe^2+^) and the oxidized ferric state (Fe^3+^), facilitating the stepwise transfer of electrons [4]. The overall reaction can be summarized as: NO_2_^−^ + 6H^+^ + 6e^−^ → NH_3_ + 2H_2_O. The electrons required for this reduction are sourced from electron donors, such as cytochrome c, which transfers electrons via the electron transport chain to the heme group in ccNiR [51]. Protons are supplied from the surrounding environment and are transported to the active site through proton channels, participating in the reduction of nitrite and its intermediates.

Despite catalyzing the same overall transformation, ccNiR and fdNiR diverge markedly in both structure and mechanism [52,53]. ccNiR harbors a decaheme c-type heme scaffold that not only shuttles electrons efficiently but also orchestrates tight proton coupling through an intricate network of hydrogen bonds and proton channels [46]. In contrast, fdNiR relies on a single siroheme cofactor paired with a [4Fe–4S] cluster to mediate its sequential 6-electron reduction of nitrite [47]. These distinct active-site architectures suit each enzyme to its physiological niche: ccNiR thrives under anaerobic conditions as part of bacterial respiratory chains, whereas fdNiR operates in oxygen-rich, photosynthetic contexts to assimilate nitrogen [52,53]. Their catalytic efficiencies are similarly governed by environmental cues. A steady supply of electron donors—whether cytochrome c for ccNiR or ferredoxin for fdNiR—is essential to keep the iron center in its reduced Fe(II) state; electron scarcity slows turnover [51]. Likewise, an acidic milieu enhances nitrite protonation, weakening the N–O bond and accelerating reduction, a process further enabled in ccNiR by its proton-relay pathways [54]. Finally, while ccNiR is optimally active below −120 mV and is readily outcompeted by oxygen for electrons under aerobic conditions, fdNiR’s localization within plant and algal organelles renders it far less sensitive to oxygen inhibition—ensuring seamless ammonia production across diverse redox landscapes[52,53].

## NiR Artificial Mimics

In the past decades, scientists have explored various strategies and materials to develop efficient catalysts that replicate the activity of NiR. The primary types of mimics include de novo designed proteins, reverse protein engineering-based models, bioinspired metal complexes, MOFs, and nanozymes, as listed in Table 2. These mimics offer a diverse array of options and effective catalytic solutions for studying and applying NiR, each achieved through unique design approaches and structural optimizations [55,56].

**Table 2.** Types, active centers, activities, and applications of NiR mimics

NiR mimics		Active sites	Reactions^a^	Activities	Applications	Ref.
CuNiR mimicking metallopeptides^b^	Cu(II)(TRIL23H)_3_^2+^	Cu(II)	Ascorbate-associated catalysis: NO_2_^–^ + H_2_A → NO + DHA + H_2_O	Rate = 5.2 × 10^−4^ s^−1^	Designing efficient metallopetides with CuNiR activity.	[59]
	Cu(II)(TRI-H)_3_			Rate = 2.20 × 10^−6^ M/min		[154]
	Cu(II)TRIW-δmH			Rate = 0.12 s^−1^; *V*_max_ = 1.5 × 10^5^ M s^−1^; *K*_m_ = 0.18 M		[61]
	Cu(I)GRα3DH3	Cu(I)		Rate = 8.1 × 10^−3^ s^−1^		[60]
*Rp*NiR^c^	3-domain *Rp*NiR	Cu(II)		*k*_cat_ = 1.1 s^−1^; *K*_m_ = 1.6 × 10^−3^ mM	Understanding the functions of different domains of NiR and engineering new NiRs.	[64]
	2-domain *Ax*NiR			*k*_cat_ = 202.7 s^−1^; *K*_m_ = 2.1 × 10^−2^ mM		
	*Rp*NiR-core			*k*_cat_ = 2.3 s^−1^; *K*_m_ = 4.1 × 10^−1^ mM		
	Y323F *Rp*NiR-core			*k*_cat_ = 2.6 s^−1^; *K*_m_ = 2.9 × 10^−1^ mM		
*Br*NiR^c^	4-domain *Br*NiR	Cu(II)		Rate *=* 9.38 nmol s^−1^		[63]
	D439N *Br*NiR			Rate *=* 0.08 nmol s^−1^		
	DCytc *Br*NiR			Rate *=* 1.67 nmol s^−1^		
Copper complexes	Complex [**4**-H_2_O]^+^	Cu(II)	Electrocatalysis: NO_2_^−^ + 2H^+^ + e^–^ → NO + H_2_O	FE = 97.3%; *V*_max_ = 14.7 nmol s^−1^ cm^−2^; *K*_m_ = 11 mM	Understanding the mechanism of nitrite reductase enzymes and designing efficient NiR mimics.	[67]
	Cu(TMPA)			Rate constant (*k*_obs_) = 9.0 × 10^2^ s^−1^		[68]
	C_15_H_12_CuN_5_O_4_P		Ascorbate-associated catalysis: NO_2_^–^ + H_2_A → NO + DHA + H_2_O	Rate = 30.7 μmol s^−1^		[70]
	(PPN)_2_[Cu_3_(*μ_3_*-O)(μ-pz)_3_(ONO)_3_]	Cu(II)	PhSH-associated catalysis: NO_2_^–^ + PhSH → NO + OH^–^ + 1/2PhSSPh	NO yield = 97%		[155]
Fe complexes	FeN_5_H_2_	Fe(III)	Electrocatalysis: NO_2_^–^ + 8H^+^ + 6e^–^ → NH_4_^+^ + 2H_2_O	FE > 90%, TON = 65	Eco-friendly production of ammonia and removal of nitrate/nitrite in wastewater.	[71]
	Fe(TPPS)			Conversion = 70%		[156]
	PEDOT–BIPY–Fe			FE = 90%		[157]
	[N(afaCy)3FeOTf]OTf (1-Cy)	Fe(II)	1,2-diphenylhydrazine-associated catalysis: NO_2_^−^ + 2H^+^ + e^–^ → NO + H_2_O	TON = 1.9	This sequence mirrored the proposed mechanism of nitrite reduction in biological systems, where the distal histidine residue shuttles protons to the active site.	[158]
	[N(afaMes)3Fe(OTf)]OTf (1-Mes)			TON = 4.0		
	Fe-SAPS1	Fe(III)	4-nitrophenol → 4-aminophenol	*k* = 0.047 min^−1^	Using Fe-NiR mimics for the catalyzing the reduction of nitro to amino groups.	[75]
	FeTSPP			*k* = 0.048 min^−1^		
	FeTMPyP			*k* = 0.068 min^−1^		
MOFs	Zr-BTB MOF@Hemin-THBA	Fe(III)	Electrocatalysis: NO_2_^–^ + 7H^+^ + 6e^–^ → NH_3_ + 2H_2_O	FE = 83%	Eco-friendly and energy-saving production of ammonia.	[78]
	Zr-BTB@Hemin			FE = 58%		
	Cu-BDC nanosheets	Cu(II)/Cu(I)	Electrocatalysis: NO_2_^−^ + 2H^+^ + e^–^→ NO + H_2_O	FE = 62%	Antibacterial coatings	[77]
Nanozymes	Cu nanowire array	Cu(II)/Cu(I)/Cu(0)	Electrocatalysis: NO_3_^−^ → NO_2_^−^ → NH_3_	FE = 91.5% (NO_3_^−^ → NO_2_^−^) FE = 100% (NO_2_^−^ → NH_3_)	Eco-friendly and energy-saving production of ammonia	[88]
	CuCo bimetallic catalyst	Cu(0)/Co(0)		FE = 100%		[89]
	Cu nanowire	Cu(0)	Electrocatalysis: NO_2_^–^ + 7H^+^ + 6e^–^ → NH_3_ + 2H_2_O	FE = 100%		[87]
	Cu*_x_*Ir_(100−*x*)_ alloy NPs	Cu(0)/Ir(0)		Activity = 19.1 mmol _surface atom_^−1^ min^−1^; NH_3_ selectivity = 100%		[90]
	Pd NPs	Pd(0)		Activity = 23.5 mmol _surface atom_^−1^ min^−1^; NH_3_ selectivity <0.07%		
	FeP nanoarrays	Fe(III)		FE = 83%		[159]
	MoS_2_ nanosheets	Mo(IV)	NO_2_^−^ + 2H^+^ + e^–^ → NO + H_2_O	NA	Antibacterial applications	[94]
	CuS/NFLA NHs	Cu(II)/Cu(I)	Ascorbate-associated catalysis: NO_2_^–^ + H_2_A → NO + DHA + H_2_O	*V*_max_ = 3.0 × 10^−8^ M s^−1^; *K*_m_ = 31.4 mM		[95]
	Fe SAC	Fe(III)	Electrocatalysis: NO_2_^−^ + 2H^+^ + e^–^→ NO + H_2_O	NO rate = 1.5 μM (min μg) ^−1^; FE_NO_ = 20%		[98]
	Cu(II)Me_3_TACN	Cu(II)	NO_2_^−^ → NO	NO yield = 93%	Inhaled NO therapy and for use in cardiopulmonary bypass surgery	[108]
	AuNRs-WS_2_	Au(0)		*K*_m_ = 42.48 mmol/l	For the reduction of TCA and nitrite	[128]
	CuCP-MWCNTs-SPCE	Cu(II)/Cu(I)	NO_2_^−^ → NO	Reproducibility = 1.73%	Effectively detecting nitrite concentrations ranging in standard and human saliva samples	[132]

### De novo designed and reverse protein engineered mimics

**Fig. 4.**
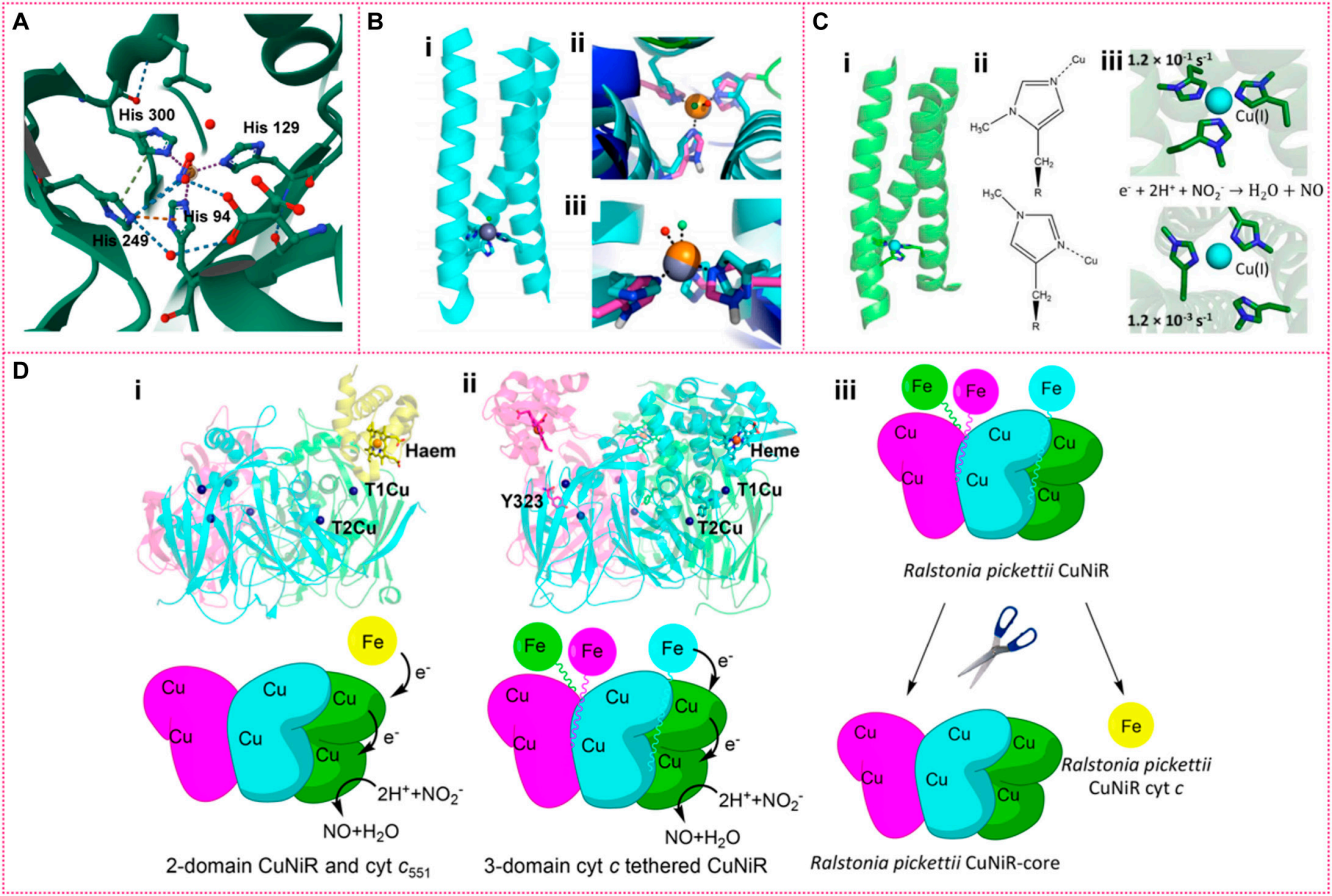
De novo protein design reverse protein engineering-based mimics. (A) T2 Cu center of CuNiR. (B) Model of metallopeptide Cu(I/II)(TRIL23H)_3_^+/2+^ (i), view of the Zn(II)(H_2_O)(His)_3_ site (ii), side view of the 2 metal sites in ii (iii). Reprinted with permission from [59]. Copyright (2012) *Proceedings of the National Academy of Sciences of the United States of America*. (C) Model of Cu(I)(TRIW-H)_3_ (i); chemical structures of (top) _δm_His(N(pros)-methyl-l-histidine) and (bottom) _εm_His (N(tau)-methyl-l-histidine) (ii); models of the metal binding sites of (top) Cu(I)(TRIW-_δm_H)_3_ and (bottom) Cu(I)(TRIW-_εm_H)_3_ (iii). Reprinted with permission from [61]. Copyright © 2019 American Chemical Society. (D) Strategy for studying the tethering of redox partners in CuNiRs. (i) Structure (top) and mechanism (bottom) of the 2-domain AxNiR. (ii) Structure (top) and mechanism (bottom) of the 3-domain CuNiR RpNiR. In (i) and (ii), the 3 monomers of the trimeric CuNiRs are shown in green, magenta, and cyan, and cytochrome c551 is shown in yellow. (iii) Strategy for dissecting the 3-domain cytochrome c-tethered *Ralstonia pickettii* CuNiR into its component domains, with colors as indicated above. Reprinted with permission from [64]. Copyright © 2019 American Chemical Society.

De novo protein design is a method for creating proteins from scratch, where researchers employ computational and experimental techniques to design proteins with specific structures and functions, independent of existing natural proteins. This approach enables scientists to generate entirely new proteins, overcoming the limitations of current protein structures to achieve targeted functional goals [57,58]. For instance, Pecoraro’s group designed a metal peptide, Cu(I/II)(TRIL23H)_3_^2+^/^3+^, that mimics the T2 copper active center of natural CuNiR (Fig. 4A) [59]. In this design, Cu(I) ions are embedded within an α-helix-coiled structure, coordinated by 3 histidine residues, while Cu(II) ions are coordinated by 3 histidines and 1 or 2 water molecules (Fig. 4B). This engineered peptide demonstrates promising catalytic activity, effectively reducing nitrite to NO. At pH 5.8, it maintains high efficiency across multiple catalytic cycles. Additionally, another researcher group designed a simple CuHis₃ binding site (GRα3D H3) within an antiparallel 3-helix bundle scaffold, where 3 histidine residues coordinate with the copper ion [Cu(I)]. This CuHis₃ binding site shows enhanced catalytic activity in the nitrite reduction reaction, with further optimization increasing its catalytic activity by 18-fold compared to other design structures (TRIW-H) [60]. Mutating the hydrophobic layers above or below the CuHis₃ binding site to Asp or Ala in the second coordination sphere can remarkably enhance NiR efficiency. Therefore, Pecoraro et al. further presented de novo designed CuNiRs incorporating noncanonical amino acids, 1-methyl-histidine and 3-methyl-histidine, to enforce δ- or ε-nitrogen ligation via methylation (Fig. 4C). This approach allowed direct comparison of the 2 ligation states within the same protein fold, showing that ε-nitrogen ligation enhances nitrite reduction, with a catalytic activity 2 orders of magnitude higher than the δ-nitrogen ligated variant [61]. These studies demonstrate that de novo protein design, grounded in a deep understanding of fundamental interactions, can effectively modulate the redox properties and NiR activities of a T2 copper center.

Reverse protein engineering is a method that involves deriving the amino acid sequence of a protein based on its existing structure and function, or by analyzing and modifying its structure and function to achieve specific application goals [62]. Unlike de novo design, which starts from scratch to create new proteins, reverse engineering begins with an existing protein. Researchers identified and isolated a novel 4-domain CuNiR from *Bradyrhizobium* sp. ORS 375 (BrNiR), with its N-terminus fused to cytochrome and blue copper protein domains [63]. Through reverse engineering, they constructed a 3-domain version comparable to HdNiR and a 2-domain version analogous to known 2-dimensional (2D)-CuNiR. Although BrNiR shares 70% primary structural similarity with other 2D-CuNiRs, its catalytic activity is 20 times lower, suggesting that natural selection may have introduced subtle structural changes to adapt to different environmental needs. The reverse engineering process revealed that the addition of extra domains did not enhance catalytic activity and might even reduce efficiency [63]. Furthermore, researchers designed a 3-domain CuNiR with N-terminal fusion, incorporating cytochrome, blue copper protein, and CuNiR core domains [64] (Fig. 4D). Despite the theoretical potential of multidomain designs to improve enzyme catalytic efficiency by enhancing electron transfer rates, the practical addition of extra domains resulted in decreased catalytic efficiency. This indicates that domain linkage is not always beneficial to enzyme function and may sometimes inhibit catalytic performance. This finding underscores the importance of carefully considering domain interactions when optimizing enzyme design through reverse protein engineering.

### Biomimetic copper and iron complexes

**Fig. 5.**
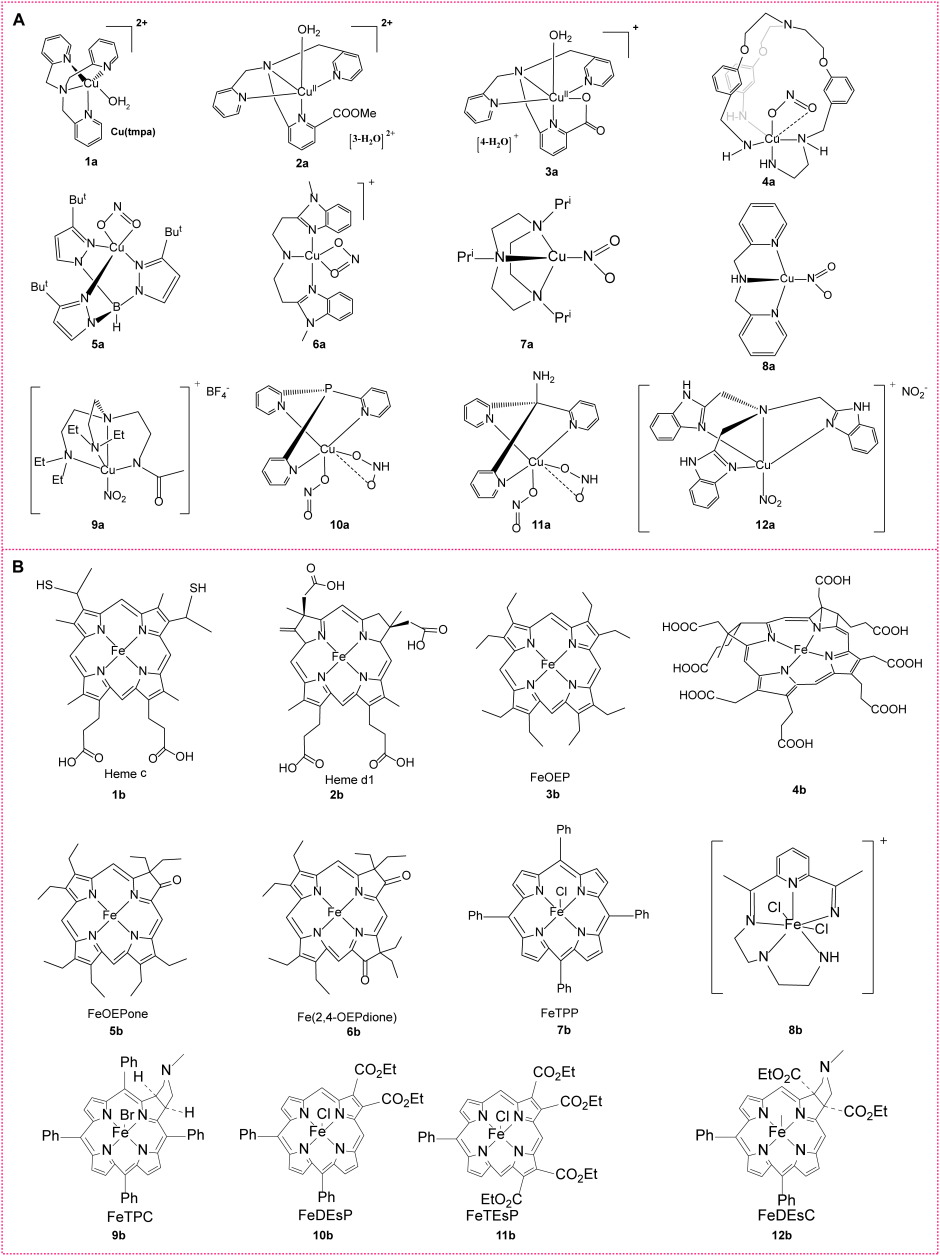
Metal complex-based NiR mimics. (A) Structures of several typical Cu-N complexes coordinated with nitrite (1a to 12a). (B) Structures of several typical iron porphyrins (1b to 12b).

**Copper complexes.** In CuNiRs, there are 2 main copper centers: Type 1 (T1) and Type 2 (T2). The T1 copper center primarily facilitates electron transfer, while the T2 copper center acts as the active site for the nitrite reduction reaction. The goal of designing CuNiR mimics is to recreate its key functions in biological catalysis, with the selection of appropriate ligands being crucial to simulate the copper coordination environment. Commonly used ligands include imidazole [65], histidine [61,66], and various nitrogen or oxygen-containing ligands. Often, nitrogen-donor chelating ligands such as bis[(1-methylbenzimidazol-2-yl)methyl]-amine [65], bis[2-(1-methylbenzimidazol-2-yl)ethyl]amine (2-BB) [65], *N*-acetyl-2-BB [65], tetradentate tris(2-methylpyridyl)amine (TMPA) [67,68], hydrotris(triazolyl)borate [69] ligands, and tridentate N-donor ligands [66] are employed (Fig. 5A). These ligands effectively mimic the histidine environment of the T2 copper center [70]. For example, the TPA ligand, with its 3 2-pyridylmethyl groups, can form stable multidentate complexes with copper ions, and its relatively rigid structure contributes to a well-defined coordination environment [66]. Additionally, varying the ligands or modifying their structures can provide insights into how these changes affect the coordination environment and catalytic activity of the copper center [66]. For instance, introducing different substituents in Cu(II) complexes can modulate the redox potential of Cu(I)/Cu(II) [70]. To confirm the 3-dimensional structures of these mimics and their coordination environments compared to natural CuNiR, researchers employ techniques such as x-ray crystallography, nuclear magnetic resonance, microanalysis, mass spectrometry, infrared spectroscopy, electrochemical studies, and DFT calculations. Finally, electrochemical, spectroscopic, and catalytic reaction experiments are conducted to verify the functionality of these mimics and assess their effectiveness in catalyzing nitrite reduction. By utilizing copper complexes to simulate the reaction center structure and function of CuNiR, these mimics not only elucidate the detailed mechanisms of nitrite reduction but also facilitate the development of simple, stable, and efficient nitrite-reducing enzymes.

**Iron complexes.** Iron-based NiRs primarily utilize iron porphyrins as their active centers. Consequently, researchers have synthesized various NiR mimics based on iron porphyrins (Fig. 5B). The catalytic activity of these iron porphyrins and their analogs closely depend on their molecular structure. By finely tuning the coordination environment, electronic structure, and group modifications of iron porphyrins, their catalytic performance can be obviously enhanced.

1. *Coordination environment design and catalytic activity*: The coordination environment of iron porphyrins is a crucial factor determining their catalytic activity [71,72]. Different porphyrin macrocyclic structures exhibit varying catalytic performances due to the distinct effects these structures have on the iron center’s coordination environment. For instance, variations between heme c and sulfoheme can lead to changes in the electronic density and reduction potential of the iron center, thereby affecting nitrite reduction activity [73]. By synthesizing model complexes with similar structures, researchers can validate how fine-tuning these coordination environments impacts catalytic reactions, further simulating the natural functions of NiRs.
2. *Electronic structure adjustment and catalytic efficiency*: Adjusting the electronic structure of iron porphyrins is a key method for optimizing their catalytic performance. For example, introducing electron-withdrawing groups (such as halogens or nitro groups) onto the porphyrin macrocycle can reduce the electronic density at the iron center, enhancing its ability to reduce nitrite [74,75]. This adjustment directly influences the selectivity and Faradaic efficiency (FE) of iron porphyrins in the electrocatalytic reduction of nitrite. Research indicates that the reduction potential of iron porphyrins can be precisely tuned by these groups, optimizing their catalytic performance and remarkably enhancing reaction rates and selectivity.
3. *Effect of group modifications on catalytic reactions*: Group modifications, particularly the introduction of functional groups such as guanidyl groups, can remarkably enhance the catalytic activity of iron porphyrins. For instance, iron porphyrin complexes modified with guanidyl groups exhibit accelerated nitrite reduction reactions through proton transfer facilitated by these groups. This design mimics the proton transfer processes in natural NiRs and enables efficient reactions without external proton sources, demonstrating the critical role of guanidyl groups in the catalytic process [74]. Additionally, guanidyl modifications improve the selectivity and reaction rate of the complexes in NO generation, underscoring the importance of group modifications in optimizing catalytic performance.
4. *Structural modification and catalytic enhancement in nanomaterials*: Research has shown that loading iron(III) porphyrins onto nanostructured materials can remarkably enhance their catalytic activity for the 6-electron reduction of nitrite to ammonia [75]. Using ion self-assembly methods, researchers have prepared nanostructured materials with varying charges, which exhibit notable temperature dependence and light responsiveness in catalytic performance. These nanostructures not only improve the stability of iron porphyrins but also enhance their catalytic activity in reduction reactions. This example illustrates that incorporating nanomaterials can effectively boost the catalytic performance of iron porphyrin-based compounds.

The catalytic activity of iron porphyrins and their analogs is profoundly influenced by factors such as coordination environment, electronic structure, and group modifications. By meticulously adjusting these molecular features, researchers can effectively optimize catalytic performance and mimic the natural functions of NiR. These studies deepen our understanding of enzyme catalysis mechanisms and provide important guidance for developing new, highly efficient catalysts.

### Biomimetic MOFs

**Fig. 6.**
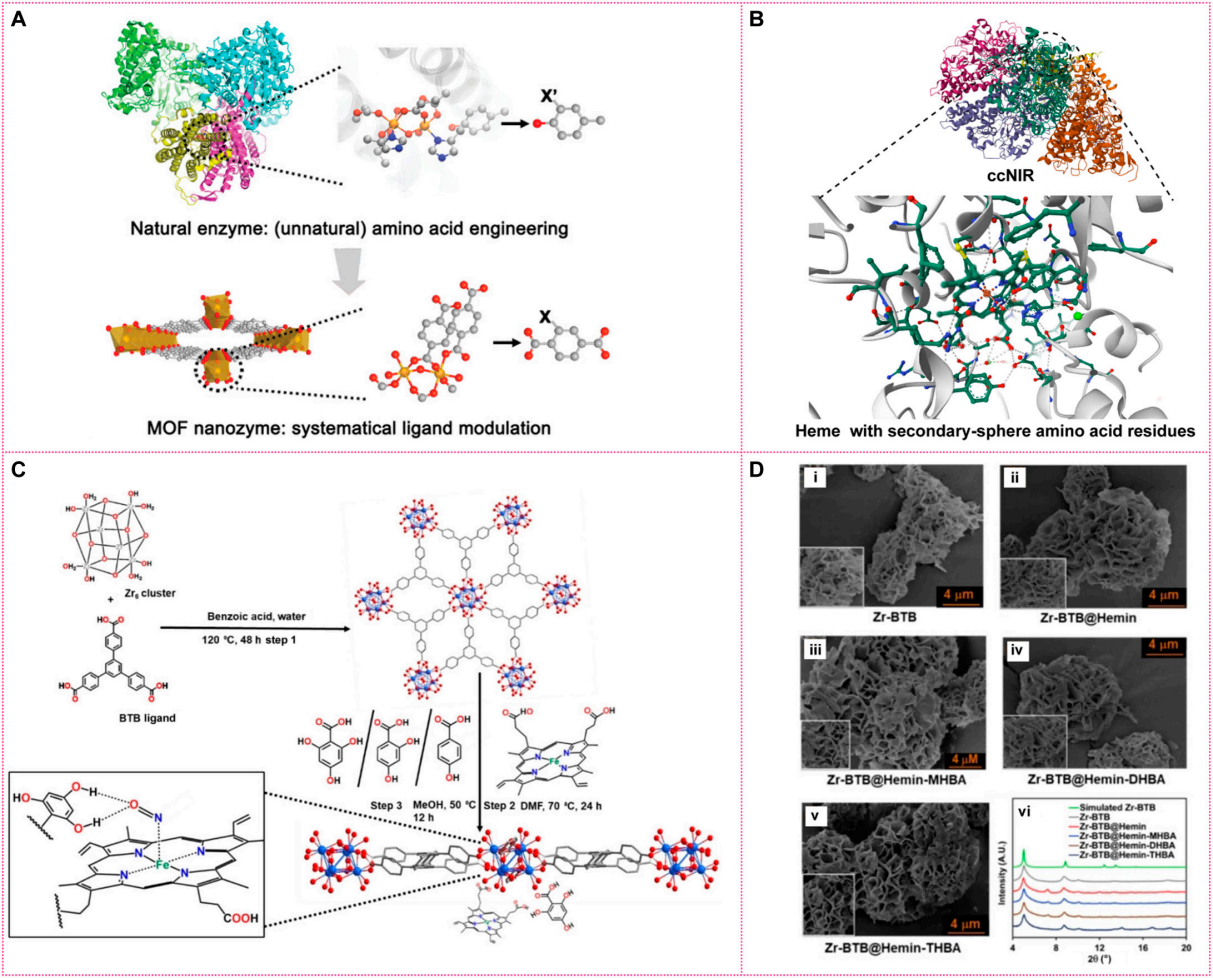
Preparation methods and morphologies of MOF-based NiRs. (A) Protein-engineering-inspired MOF nanozyme modulation. Reprinted with permission [76]. Copyright © 2020 Wiley-VCH GmbH. (B) illustration of ccNiR and its heme group with secondary sphere amino acid residues. (C) Schematic illustration of the synthesis of Zr-BTB MOF with the post-synthetic modification by heme and hydroxybenzoic acids. (D) SEM images of Zr-BTB (i), Zr-BTB@Hemin (ii), Zr-BTB@Hemin-MHBA (iii), Zr-BTB@Hemin-DHBA (iv), Zr-BTB@Hemin-THBA (v), and XRD patterns of different BTB@Hemin hybrids (vi). Reprinted with permission [78]. Copyright © 2024 The authors. *Angewandte Chemie International Edition* published by Wiley-VCH GmbH.

Taking the merit of protein engineering, the primary and secondary coordination spheres of metalloenzymes can be well clarified. MOFs are 3D networks of metal ions linked by multidentate organic linkers. As shown in (Fig. 6A), through rational modulation, MOF nanozymes with similar coordination and activity to metalloenzymes can be designed [76]. Inspired by this, Wang et al. [77] synthesized a Cu-BDC catalyst (BDC: benzene-1,4-dicarboxylic acid) with coordinated Cu(II) sites, which serves as a heterogeneous electrocatalyst for controlling nitrite reduction to NO for catheter antibacterial applications. Hod et al. found that, besides the heme cofactors, the secondary-sphere amino acid residues like histidine (His), arginine (Arg), and tyrosine (Tyr) along with axial lysine (Lys) ligand of NiR (e.g., ccNiR; Fig. 6B) are also important in the nitrite activation step. To mimic this secondary-sphere proton-translocation and H-bond donating ability of Tyr residues in native NiR enzyme, they designed a series of heme (Fe-porphyrin)-based MOFs with different phenolic ligands tethered within the MOF (Fig. 6C), showing a flower like morphology with typical 2D-layered nanosheets (Fig. 6D), which displayed marked enhancement of both catalytic NH_3_ selectivity (>90%) and rate (by ~5 times) [78]. Moreover, by introducing functional groups or metal clusters into MOF pores, researchers can precisely regulate the catalytic active centers’ microenvironment. Lu’s group utilized vapor deposition to introduce iron ions into the MOF NU-1000 structure, followed by the addition of dodecanethiol to form iron-thiolate clusters. These clusters effectively mimic NiR’s structural characteristics, efficiently reducing NO₂^−^ to ammonia under physiological pH conditions [79]. Furthermore, MOFs generally exhibit high chemical stability, retaining catalytic performance even under harsh conditions. By leveraging their unique structure and chemical properties, MOF-mimicking NiRs enable efficient electrocatalytic nitrite reduction through careful structural design and functionalization.

### NiR nanozymes

Nanozymes are nanomaterials that exhibit catalytic activity similar to natural enzymes [80–82]. Composed of metals, metal oxides, carbon-based materials, or other inorganic compounds, these nanomaterials mimic the active centers of enzymes and catalyze a variety of biochemical reactions [83]. Nanozymes possess distinctive physical and chemical features, such as high surface area, varied electronic structures, and tunable active sites, allowing them to perform efficiently in catalytic reactions. Recent advances include the development of copper nanowires (NWs), copper–cobalt bimetals, copper–iridium, copper sulfide, molybdenum sulfide, and single-atom iron catalysts, which utilize copper, molybdenum, and iron as active centers to demonstrate promising NiR activity[84–86].

**Fig. 7.**
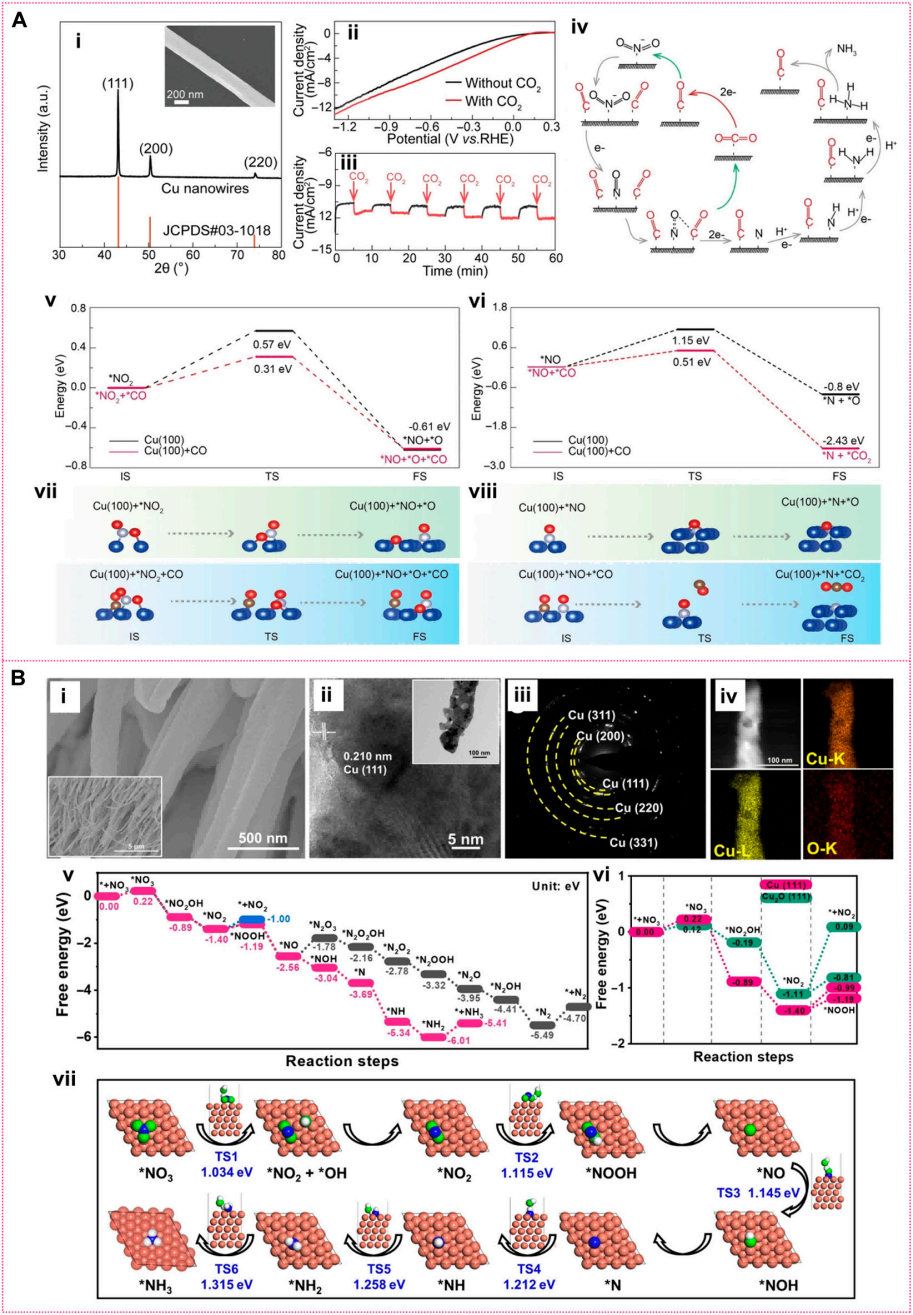
Morphology and structural characterizations of Cu NWs with corresponding catalytic mechanism. (A) SEM image and XRD pattern of copper nanowires (Cu NWs) (i); linear sweep voltammetry of nitrite electroreduction on Cu NWs (ii); chronoamperometry of Cu NWs under intermittent CO_2_ (iii); possible reaction pathway of NO_2_^–^ reduction to NH_3_ in the presence of CO_2_ (iv); energy barriers and structures of NO_2_deoxygenation (v and vii); energy barriers and structures of NO deoxygenation (vi and viii). Reprinted with permission from [87]. Copyright © 2022 Wiley-VCH GmbH. (B) SEM images at 2 scales (i), HRTEM and TEM (inset) images (ii), SAED pattern (iii), and STEM and elemental mapping (iv) of Cu-RD-KOH; reaction free energy of different intermediates for NO_3_^−^ reduction reaction on Cu (111) surface of Cu-RD-KOH (v); the comparison of the free energy diagram for the reduction of NO_3_^−^ into NO_2_^−^ and *NOOH over Cu (111) and Cu_2_O (111) at an applied potential of U = 0 V (vi). Simulated atomistic structure scheme shows a reaction pathway for the reduction of NO_3_^−^ to NH_3_ and the corresponding transition state (TS) energy barriers (vii). Reprinted with permission from [88]. Copyright © 2023 The authors. *Angewandte Chemie International Edition* published by Wiley-VCH GmbH.

**Metallic copper-based nanozymes**. Copper nanostructures, particularly copper NWs [Fig. 7A(i)] [87], exhibit high catalytic activity in nitrite reduction due to their large surface area and high aspect ratio, which provide abundant active sites. This catalytic performance is especially enhanced by 3.5 times in the presence of carbon dioxide (CO_2_) [Figure 7A(ii and iii)], due to lowering the activation energy for nitrite reduction to ammonia [87]. Based on the DFT calculations [Fig. 7A(v to viii)] and in situ FTIR measurements, the reaction pathway of nitrite reduction and the influence of CO_2_ over copper NWs shown in Fig. 7A(iv) can be provided. CO_2_ was first reduced to the *CO species, where * represents Cu NWs. It could reduce the *NO intermediates to *N, promoting the rate-determining step (deoxygenation of *NO intermediates) of nitrite electroreduction through decreasing the reaction energy barrier. In this process, the *CO was oxidized by *NO to CO_2_ and completes the catalytic cycle. In addition, the adsorbed *CO species could accelerate the hydrogenation of *NH_2_ intermediates to NH_3_ [87]. Interestingly, when building Cu NW arrays [Fig. 7B(i to iv)], they can mimic the 2-stage bacterial nitrate respiration process: nitrate is first converted to nitrite by nitrate reductases (NRases), then reduced to NO or ammonium by NiRs. This process follows an indirect electrocatalytic nitrate reduction reaction (NitrRR) with a [2+6]-electron pathway for both nitrite (+0.2 V vs. RHE) and ammonia (+0.1 V vs. RHE) synthesis [Fig. 7B(v to vii)], achieving FEs of 91.5% and 100%, respectively [88]. Introducing additional metals to create multimetallic nanozymes can further boost catalytic performance due to the synergistic interaction between different metal components. For instance, copper–cobalt (Cu–Co) bimetallic nanosheets, synthesized via solvothermal methods, have a layered structure with many exposed active sites, enhancing electron and proton transfer and delivering high nitrite reduction performance [89]. In these nanosheets, cobalt donates electrons and protons to enhance electron transfer, while copper provides adsorption and binding sites for NO*_x_*^−^, promoting efficient NO_2_^−^ reduction. The combined activity of copper and cobalt remarkably boosts catalytic efficiency [89]. In the case of the alloyed copper–iridium (Cu–Ir) NPs, with high surface area and favorable alloy effects, they exhibit nearly 100% selectivity for ammonia in nitrite reduction [90]. This high selectivity is attributed to the synergistic effect between copper and iridium, where copper is mainly responsible for nitrite adsorption and initial reduction, while iridium improves ammonia production by lowering the reaction’s activation energy [90]. Drawing inspiration from Mo-NRases and Fe-ccNiR for nitrate and nitrite reduction in nature, Mougel et al. [91] developed a Fe/Mo bimetallic MXene (Mo_2_CT_x_: Fe) electrocatalyst. The Fe-Mo synergy enabled an FE of 41% and an NH₃ yield rate of 3.2 μmol h^−1^ mg^−1^ in acidic media, and 70% FE with 12.9 μmol h^−1^ mg^−1^ in neutral media for NO_3_^−^ to NH_4_^+^ reduction.

**Fig. 8.**
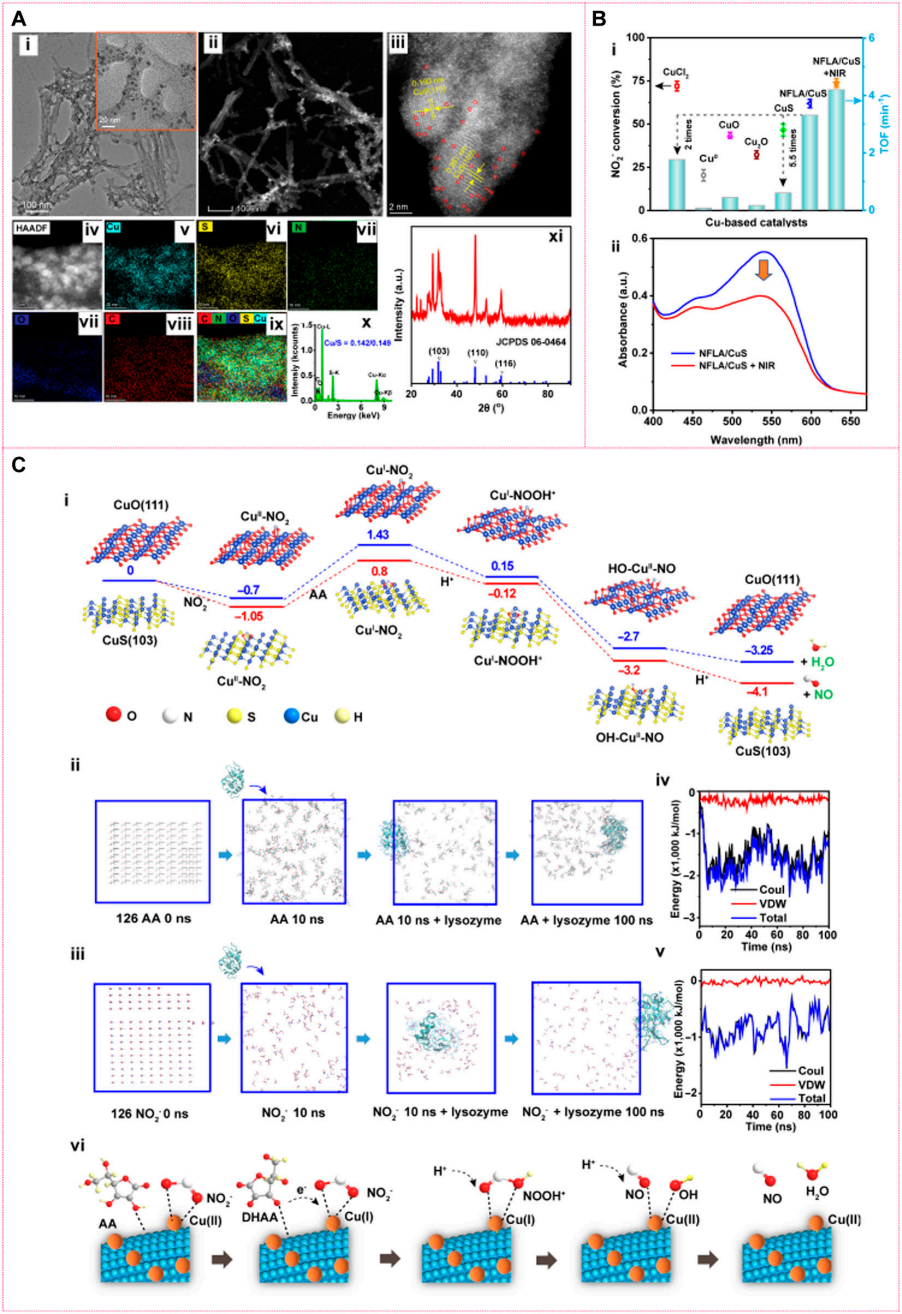
Morphology, catalytic activity, and catalytic mechanism of NFLA/Cu NHs (nanofibrous lysozyme assemblies [NFLA]). (A) TEM images of NFLA/CuS NHs (i to iv) and corresponding element maps (v to ix) and element distribution (x) of NFLA/CuS NHs, XRD pattern of NFLA/CuS NHs (xi). (B) Catalytic activity of NFLA/Cu NHs compared with other copper-based materials (i), and catalytic activity enhanced by light irradiation (ii). (C) Energy diagram for the reduction of NO_2_^−^ to NO catalyzed by the CuS (103) plane and Cu (111) plane (i); schematic diagram of the molecular dynamics (MD) simulation for the interaction between lysozyme and AA (ii); schematic diagram of the MD simulation for the interaction between lysozyme and nitrite (iii); diagrams of the interaction energy variation over time for lysozyme binding to AA (iv); diagrams of the interaction energy variation over time for lysozyme binding nitrite. Mole ratio (v); proposed mechanism scheme of the catalytic reduction of NO_2_^−^ to NO by NFLA/CuS NHs in the presence of AA (vi). Reprinted with permission from [95]. Copyright © 2024 The authors. *Science Advances* published by the American Association for the Advancement of Science.

**Metal sulfide-based nanozymes**. Metal sulfides, such as molybdenum disulfide (MoS_2_) and copper sulfide (CuS), possess multivalent active centers and good electron transport properties. As nanozymes, they enhance substrate adsorption and accelerate electron transfer, thereby improving catalytic efficiency [92,93]. MoS_2_ nanosheets, for example, prepared via hydrothermal or liquid-phase exfoliation methods, feature a layered structure with abundant active edge sites, facilitating reactant adsorption and activation. This enables the efficient conversion of nitrite (NaNO_2_) to NO, achieving remarkable antibacterial effects [94]. To improve the nitrite reduction activity, Feng et al. have recently developed a bioinspired strategy to synthesize a bio-inorganic hybrid nanozyme (NFLA/CuS), of which nanofibrous lysozyme assemblies (NFLA) mimic the framework of CuNiR while ultrasmall CuS NPs with Cu(I)/Cu(II) coexistence resemble the active sites. Figure 8A clearly demonstrates the hybrid nanostructure of NFLA/CuS, contributing a 5.5 times higher than the blank CuS catalyst and a 2.2 times higher than the homogenous Cu^2+^ ion catalyst [Fig. 8B(i)] [95]. More importantly, owing to the intrinsic near-infrared (NIR) optical absorption, using NIR irradiation can further enhance the catalytic activity [Fig. 8B(ii)], which is of great significance when applying for antibacterial or anticancer [95]. Based on the results of DFT and molecular dynamics (MD) calculation [Fig. 8C(i to iii)], a typical 5-step reaction pathway similar to that of natural CuNiR for NFLA/CuS hybrid nanozyme catalyzing nitrite to NO is proposed [Fig. 8C(iv)]. Cu(II) active sites on the CuS surface bind with NO₂^−^ to form a Cu(II)–NO_2_ complex, which is subsequently reduced by ascorbic acid (AA) to form the Cu(I)–NO_2_ intermediate. After reacting with H^+^, a Cu(I)–NOOH complex forms and dissociates into NO–Cu(II)–OH, eventually generating NO/H_2_O and regenerating Cu(II) centers [95]. In this process, the NFLA facilitate the catalytic reduction NO₂^−^ to NO due to the strong substrate (AA and NO₂^−^) of NFLA and the electron transfer between NFLA and CuS.

**Fig. 9.**
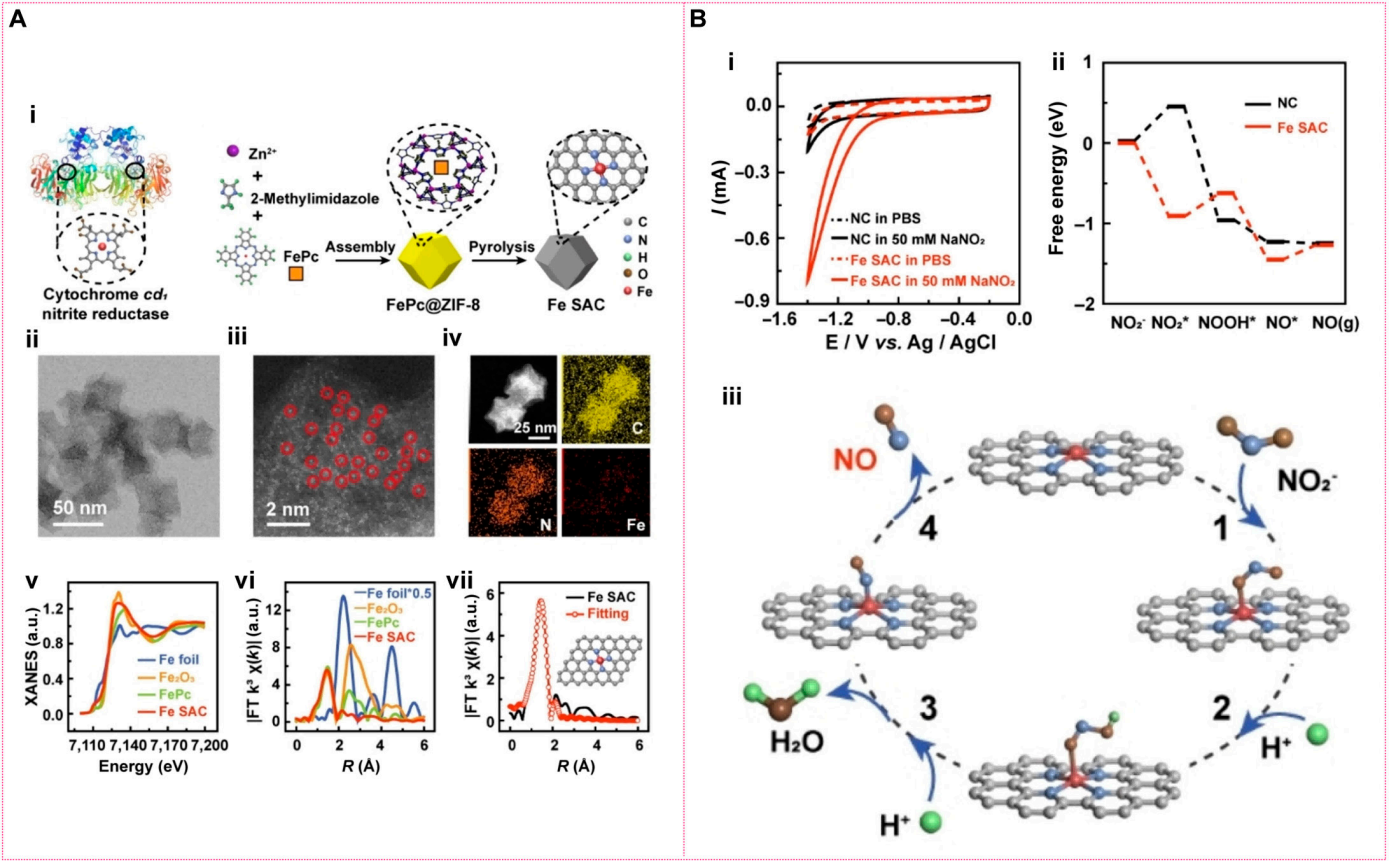
Morphology, catalytic activity, and catalytic mechanism of iron-based single-atom catalyst (Fe SAC). (A) The structure of natural cytochrome cd1 nitrite enzyme and schematic illustration of the synthesis of FeSAC (i); HAADF-STEM image of Fe SAC (ii); AC-STEM of Fe SAC (iii); HAADF-STEM image and corresponding EDX element mapping of Fe SAC (iv); XANES spectra of Fe SAC (v); Fourier transforms of the Fe K-edge of Fe SAC (vi); corresponding EXAFS fitting curves of Fe SAC at R space (vii). (B) CVs obtained at GCEs modified with Fe SAC (red) or nitrogen-doped porous carbon (NC) (black) in PBS in the absence (dash) and presence (solid) of 50 mM NaNO_2_ (i); density functional theory (DFT)-calculated free energy paths of the nitrite reduction on Fe SAC (ii); proposed structures for electrocatalytic reduction process of nitrite on Fe SAC (iii). Reprinted with permission from [98]. Copyright © 2022 Wiley-VCH GmbH.

**Single-atom nanozymes (SAzymes).** SAzmyes represent a new generation of nanozymes featuring maximized atomic utilization and precisely designed active sites, often surpassing the activity of natural enzymes [96,97]. The efficient and on-demand NO generation essentially dictates its concentration-dependent therapeutic activity. Recently, Jin et al. [98], inspired by cd1NiR, have provided a MOF-based pyrolysis method [Fig. 9A(i)] to prepare an iron-based single-atom catalyst (Fe SAC) with single iron atoms anchored on a nitrogen-doped porous carbon (NC) substrate. Figure 9A(ii to iv) well confirm the successful formation of Fe single atoms on the framework of NC. This iron–nitrogen coordination structure closely replicates the active center of cytochrome cd1 [Fig. 9A(v to vii)], thereby displaying outstanding performance in nitrite reduction with a maximum NO production rate of 2.1 μM (min μg)^−1^ [Fig. 9B(i)]. Moreover, the amount of NO can be controllably generated in a potential-dependent manner. Owing to the precise active site of Fe SAC, the electrocatalytic reduction of nitrite to NO over Fe SAC can be well clarified. The process begins with the strongly exothermic adsorption of NO₂^−^ on Fe SAC (Δ*G* = −0.91 eV), which is obviously superior to the endothermic adsorption on nitrogen-doped carbon (NC) (Δ*G* = +0.46 eV, serving as the rate-determining step for NC) [Fig. 9B(ii)] [98]. Subsequently, the adsorbed NO₂ undergoes protonation to form the intermediate NOOH*, with a Gibbs free energy decrease of 0.18 eV in Fe SAC compared to NC, further optimizing the reaction kinetics. Ultimately, NOOH* is converted to NO, which desorbs as the final product [Fig. 9B(iii)]. The single-atom iron enables an efficient catalytic cycle by reducing energy barriers in critical steps, stabilizing intermediates, and modulating the electronic structure. DFT calculations and atomic configuration analyses confirm that the superior performance of Fe SAC originates from the precise regulation of the reaction pathway by the single-atom active centers.

## NiRs and Their Mimics in Biomedicine

NiR is a class of metalloenzymes capable of catalyzing the reduction of nitrite into critical biological molecules such as NO or NH₃, participating in the nitrogen cycle and NO signaling regulation within organisms. NiRs and their biomimetic catalysts (e.g., metal complexes, metal sulfides, and iron single-atom materials) demonstrate substantial potential in biomedical fields such as cardiovascular and cerebrovascular disease treatment, antibacterial applications, and biosensing, owing to their high catalytic activity and tunability.

### Treatment of cardiovascular and metabolic diseases

In contrast to the classical L-arginine–NO pathway, the nitrite–NO pathway, mediated by NiRs such as hemoglobin, Mb, Ngb, XO, and AO, is considered a complementary mechanism to ensure NO production under ischemic or hypoxic conditions [99]. This pathway triggers downstream signaling that induces various biological responses, including hypoxic vasodilation, promotion of angiogenesis, modulation of glucose metabolism, enhanced exercise efficiency, regulation of mitochondrial function, and increased tolerance to ischemia/reperfusion (I/R) injury [100]. Studies have highlighted the therapeutic potential of nitrates and nitrites in treating conditions like myocardial infarction, stroke, systemic and pulmonary hypertension, and gastric ulcers [100,101]. For instance, dietary nitrite can lower BP [102] by relaxing vascular smooth muscles through cGMP production [103] and by converting nitrite to NO, which produces vasodilatory effects [104]. Recent research also indicates that inorganic nitrate and nitrite can alleviate kidney fibrosis associated with oxidative stress and NO deficiency [105,106]. By activating the nitrate–nitrite–NO pathway, this approach can enhance the phosphorylation of AMP-activated protein kinase (AMPK), increase AKT-mediated peroxisome proliferator-activated receptor-γ coactivator 1-α (PGC1α) activity, and help restore mitochondrial function [105]. Nitrite supplementation, particularly in the form of dietary nitrate, has shown promise in improving exercise performance through the nitrate–nitrite–NO pathway under hypoxic conditions. For example, nitrate-rich beetroot juice has been demonstrated to increase exercise endurance in chronic obstructive pulmonary disease patients requiring supplemental oxygen, suggesting broader applications in hypoxic conditions [107]. Additionally, a portable NO generator using electrochemical reduction of nitrite via a copper(II)–ligand electron transfer mediator complex has been developed for inhaled NO therapy and for use in cardiopulmonary bypass surgery [108]. Likewise, the antihypertensive effects of nitrate were reported in clinical and experimental studies. The first clinical evidence showing a BP-lowering effect of nitrate was reported by Larsen et al., who examined the effect of 3-day dietary supplementation with either sodium nitrate (at a dose of 0.1 mmol per kilogram of body weight per day) or placebo (sodium chloride, at a dose of 0.1 mmol per kilogram per day) on BP in 17 physically active, healthy volunteers. They conclude that short-term dietary supplementation with inorganic nitrate reduces diastolic BP in healthy young volunteers [109]. Subsequent studies showed that nitrate is able to not only reduce BP in healthy volunteers [110] but also promote sustained antihypertensive effects in hypertensive patients. Single-dose administration of dietary inorganic nitrate acutely reduces BP in normotensive healthy volunteers, via bioconversion to the vasodilator NO. Kapil et al. assessed whether dietary nitrate might provide sustained BP lowering in patients with hypertension. This is the first evidence of durable BP reduction with dietary nitrate supplementation in a relevant patient group. These findings suggest a role for dietary nitrate as an affordable, readily available, adjunctive treatment in the management of patients with hypertension [111].

**Fig. 10.**
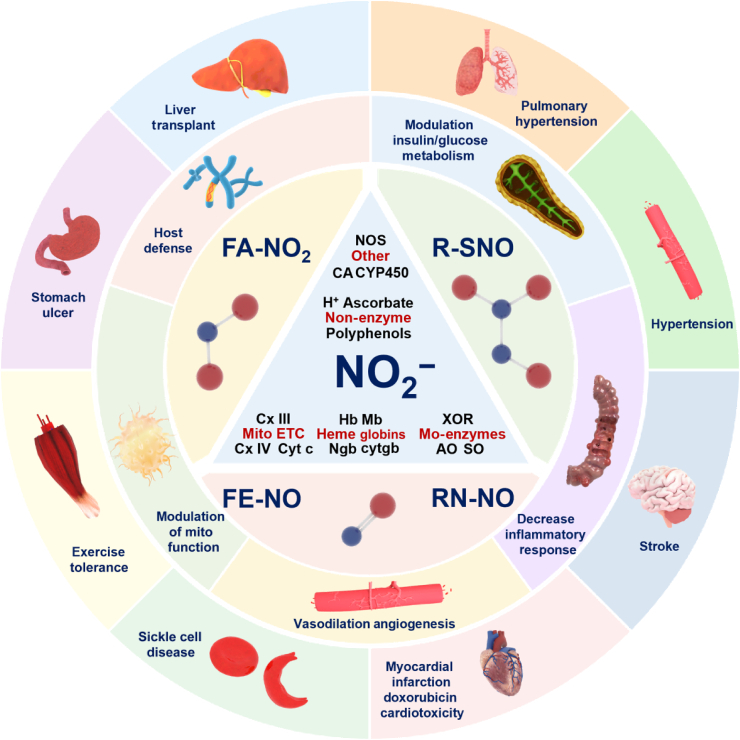
Therapeutic opportunities for nitrite reduction catalyzed by NiRs for treating cardiovascular and metabolic diseases. Nitrite reduction to NO, enhanced during hypoxia/ischemia, offers therapeutic potential. Preclinical studies show nitrite protects against ischemia–reperfusion injury, supporting its use in treating heart attacks, strokes, organ transplant issues, and sickle cell disease. It also prevents drug-induced stomach ulcers and alleviates hypertension (pulmonary/systemic) and post-hemorrhagic cerebral vasospasm via vasodilation. These multifunctional benefits highlight nitrite as a versatile therapeutic agent for oxygen-sensitive disorders.

These findings illustrate that NiRs not only play a crucial role in regulating various metabolic processes but also hold substantial therapeutic potential across a wide range of diseases (Fig. 10).

### Antibacterial therapy

**Fig. 11.**
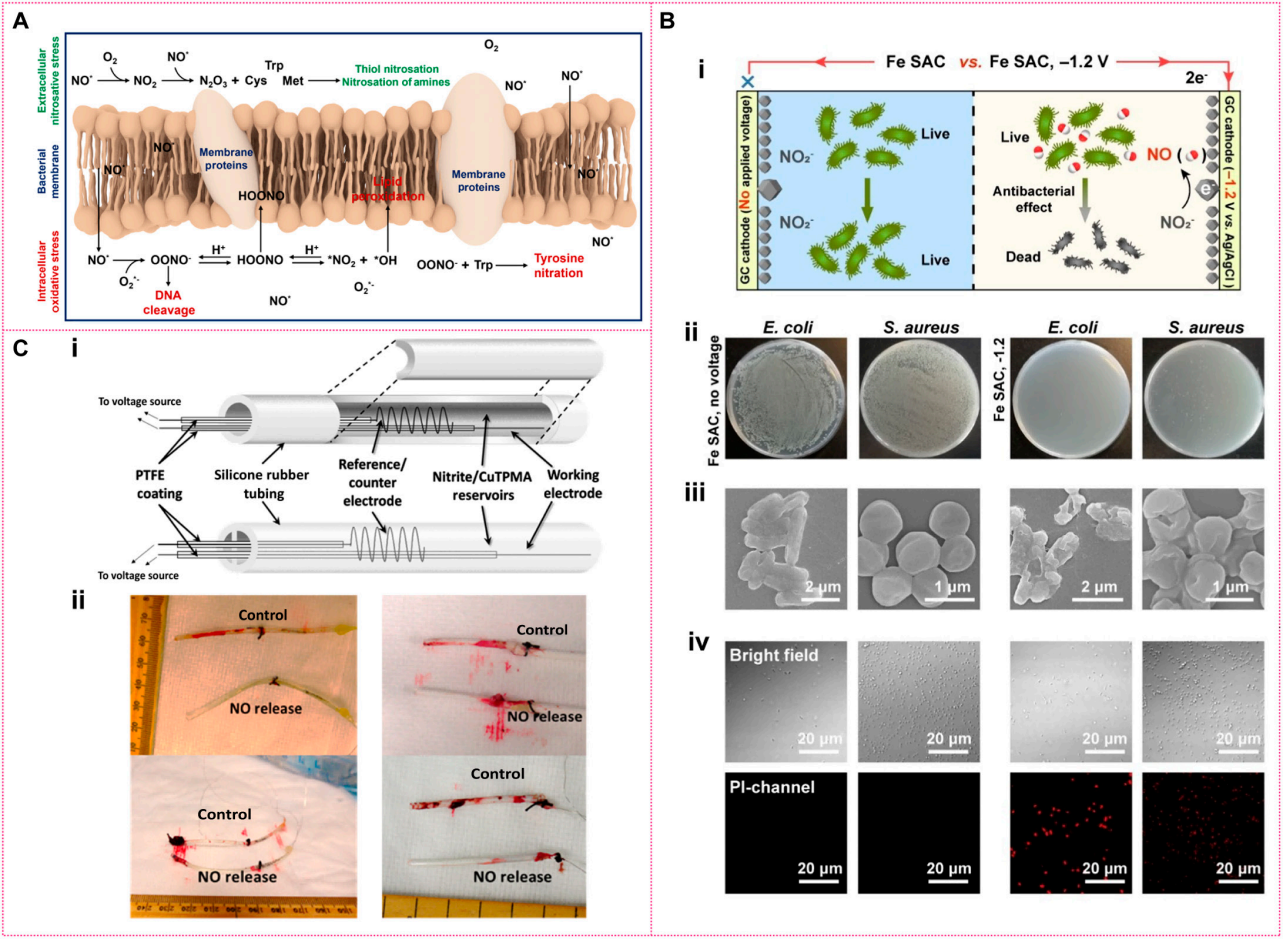
Antibacterial mechanism of NO and electrocatalytic antibacterial application. (A) Schematic illustration of mechanism of NO killing bacteria. (B) Schematic of electrochemical generation of NO for bacterial elimination (i); digital photographs of remaining bacteria inoculated agar plates (ii); SEM of *E. coli* and *S. aureus* (iii); bright field (top) and PI channel (bottle) of *E. coli* and *S. aureus* (iv). Reprinted with permission from [98]. Copyright © 2022 Wiley-VCH GmbH. (C) Schematics of single and dual lumen electrochemically modulated NO releasing catheter configurations (i); representative pictures of single (left) and dual (right) lumen catheters after removal from the vein (ii). Reprinted with permission from [116]. Copyright © 2014 American Chemical Society.

NO has demonstrated potent antibacterial properties, functioning through both oxidative and nitrosative stress mechanisms. These mechanisms induce lipid peroxidation, protein oxidation, and enzyme inactivation [112], ultimately disrupting essential bacterial processes, including cell wall integrity [113]. Additionally, NO directly damages bacterial nucleic acids through chemical reactions, impairing DNA replication and repair, which compromises bacterial survival (Fig. 11A) [95,114]. At higher concentrations, NO can inhibit biofilm dispersal through specific signaling pathways, enabling biofilm clearance in a nontoxic manner [115]. Leveraging the ability to regulate NO release through catalysis, NiR mimetics have been considered as alternative antibacterial agents to combat antimicrobial resistance. Numerous effective antibacterial NiR mimics, such as the Cu–nitrogen complex [116], Cu-MOF [77], MoS nanosheets [94], Fe SAC [98], and CuS/NFLA nanohybrids [95], have been developed for antibacterial therapy. Catalytic antibacterial therapies leveraging nitrite-mimicking enzymes can be broadly classified into electrocatalytic and thermal catalytic approaches, each tailored to distinct infection scenarios [117–120].

Electrocatalytic antibacterial therapy harnesses electric fields to drive the conversion of nitrite into highly reactive antimicrobial NO, which enables rapid pathogen eradication in superficial infections, including urinary tract infections, due to its precision, controllability, and ability to penetrate biofilms [77,98,116]. Figure 11B(i) shows the electrocatalytic system with nitrite as the precursor and Fe SAC as the NiR mimic [98]. When applying voltage for 1 h, neither *Escherichia coli* nor *Staphylococcus aureus* survive due to the sustainable generation of NO [Fig. 11B(ii)]. Scanning electron microscopy (SEM) images [Fig. 11B(iii)] clearly show that the membranes of treated bacteria become wrinkled and collapsed, which can be further confirmed by the PI staining [Fig. 11B(iv)] [98]. The results demonstrate that the generated NO is the main contributor for bacteria elimination despite the fact that the current NO generation is accompanied by the addition of NO precursor, over-reduced by-product, and pH changes. To enable the application of electrocatalytic NO generation in preventing urinary tract infections, researchers have constructed an electrocatalytic NO production system by modifying silicone rubber catheter surfaces with copper-based NiR mimics [e.g., Cu(II)TPMA complexes or copper-based MOFs] [77,116]. In a physiological nitrite-containing environment, controlled cathodic potentials were applied to drive the electrochemical reduction of nitrite into NO, achieving stable release of physiological levels of NO for over 7 days [Fig. 11C(ii)] [116]. In vitro antibacterial experiments demonstrated that daily 3-h electrocatalytic NO release reduced surface-attached viable bacteria by more than 100-fold. Short-term (7-h) in vivo experiments involving implantation in rabbit veins showed marked inhibition of thrombus formation (*P* < 0.02), along with validated biocompatibility [Fig. 11C(ii)] [116]. This strategy combines precise electrochemical regulation of NO release, low-cost fabrication, and long-lasting antibacterial/antithrombotic properties, offering a programmable solution to combat biofilm infections on medical device surfaces.

**Fig. 12.**
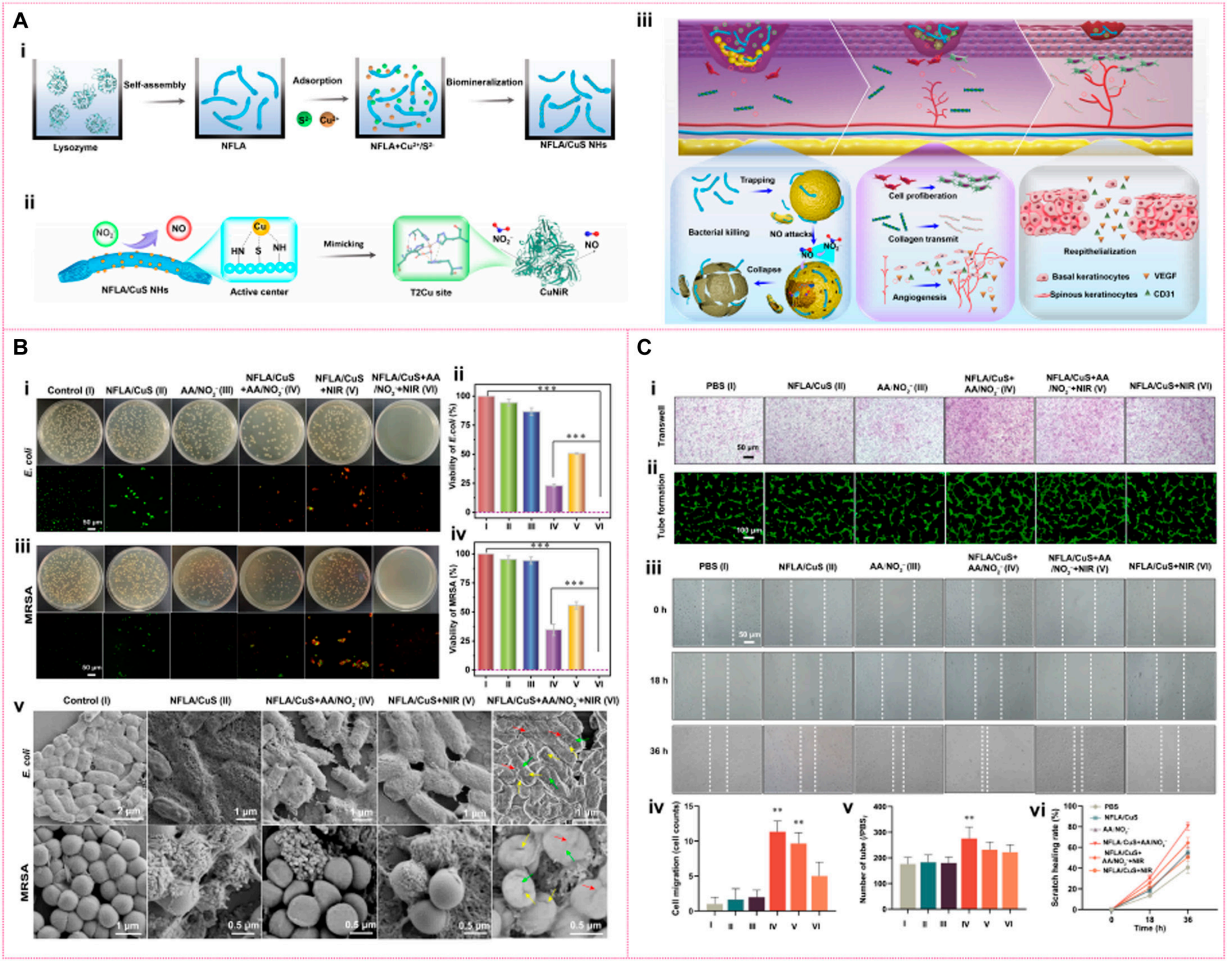
NFLA/CuS nanohybrids mimicking NiR catalysis for antibacterial therapy (A) Schematic illustration of preparation of NFLA/CuS nanostructures (i); schematic illustration of NFLA/CuS mimicking CuNiR catalysis (ii); schematic illustration of the antibacterial and tissue repair mechanism of CuNiR-like catalysis on NFLA/CuS nanostructures (iii). (B) Colony diagram and the corresponding fluorescence staining diagram of *E. coli* (i) and MRSA (ii); survival rate of *E. coli* (iii) and MRSA (iv); SEM images of *E. coli* and MRSA (v). (C) Transwell migration (i) and tube formation experiments (ii) of HUVECs; images of scratch assay of HUVECs cells after different treatments for distinct times (18 and 36 h) (iii); number of transwell migrated cells counted (iv); tube formation of HUVECs (v); healing rate of cell scratches after 36 h (vi). Reprinted with permission from [95]. Copyright © 2024 The authors. *Science Advances* published by the American Association for the Advancement of Science.

It is known that electrocatalytic NO release is obviously limited by its reliance on external devices and restricted tissue penetration. In contrast, thermal catalytic therapy—particularly enzyme-mimetic catalytic systems operable at ambient temperature[94,95]—is well-suited for treating deep chronic wound infections while promoting tissue healing. These catalytic systems leverage the structural and functional properties of nanomaterials to enhance antibacterial efficacy, either through intrinsic characteristics, structural engineering, or the integration of external stimuli such as NIR photothermal regulation to optimize reaction temperatures [83,96,121–123], thereby improving catalytic antibacterial performance. For instance, 2D MoS_2_ nanosheets with NiR-like activity not only catalyze NO generation via nitrite reduction but also induce neutrophil extracellular trap (NET) release, web-like structures of decondensed chromatin decorated with antimicrobial granular proteins (e.g., neutrophil elastase and myeloperoxidase), achieving synergistic antibacterial effects [94]. This provides a foundation for developing immune-material cooperative antibacterial strategies, such as targeted NET modulation or NO-responsive nanomaterials. Furthermore, Feng et al. [95] designed an NFLA/CuS nanohybrid with a nanofibrous network, exhibiting enhanced NiR-like activity and strong NIR photothermal properties. Harnessing the dual functionality of NO, high concentrations for bactericidal effects and low concentrations for tissue regeneration, they proposed a dynamic catalytic NO regulation strategy to integrate infection control with tissue healing (Fig. 12A) [95]. During the initial treatment phase, the nanofibrous structure entangles bacterial surfaces, ensuring localized and efficient NO delivery, while NIR-induced thermal effects enhance catalytic efficiency, intensifying bacterial disruption (Fig. 12B) [95]. In later stages, as substrate concentrations decline, sustained catalytic reactions maintain low, steady NO levels that support cell proliferation and angiogenesis (Fig. 12C), ultimately promoting effective chronic wound healing [95]. These findings highlight the potential of mimicking NiR-mediated NO catalysis, combined with the structural advantages of nanozymes, as a promising strategy for antibacterial therapy and combating bacterial resistance.

### Biosensing and detection

**Fig. 13.**
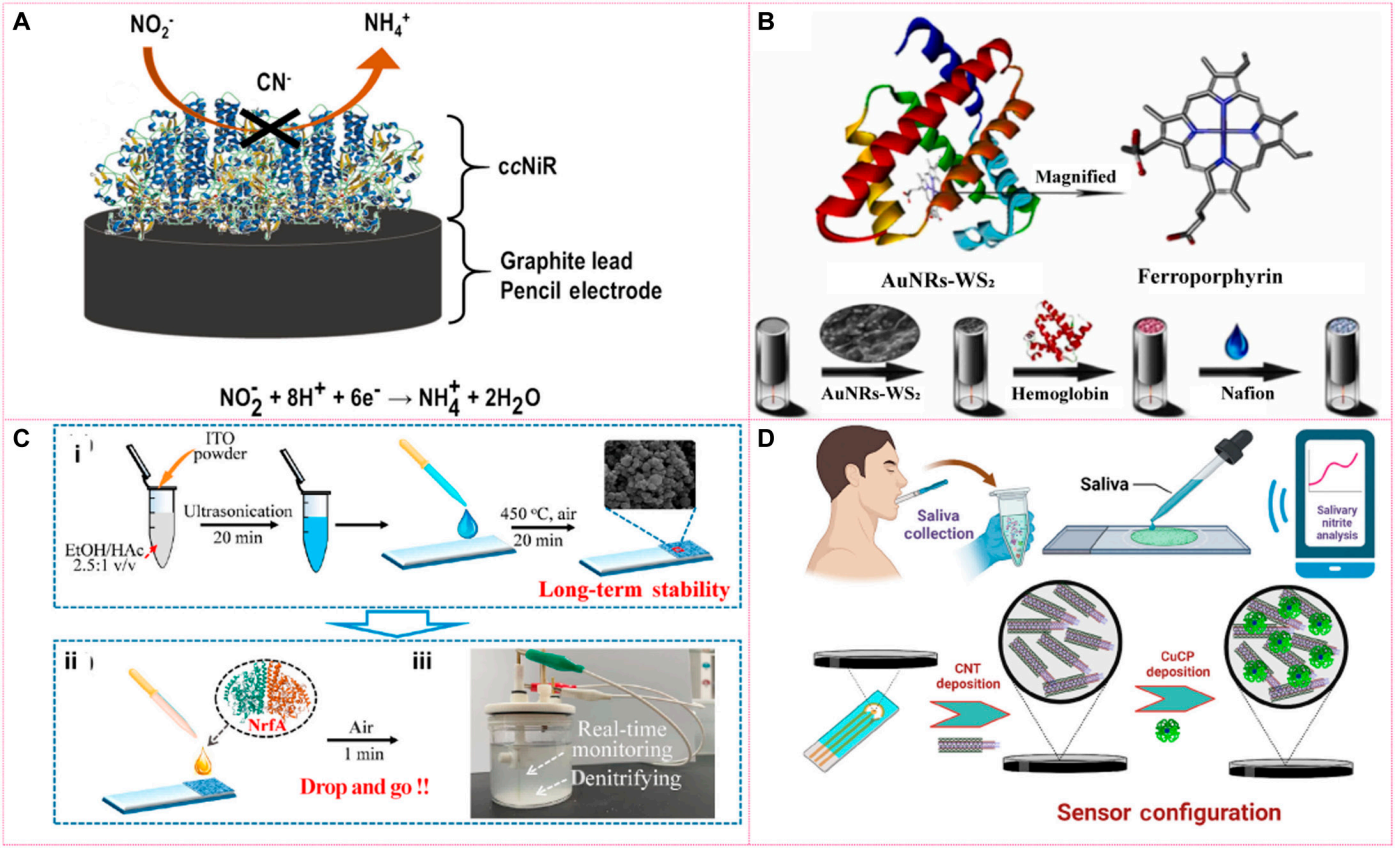
Schematic illustration of the preparation of NiR-based biosensors. (A) Multiheme ccNiR immobilized on commercial graphite pencil leads. Reprinted with permission [127]. Copyright © 2021 Elsevier B.V. (B) Hemoglobin (Hb) immobilized on AuNRs-WS_2_/CILE electrode. Reprinted with permission [128]. Copyright © 2023 The authors. Published by Elsevier B.V. (C) MesoITO (mesoporous indium tin oxide) electrode fabrication (i), NrfA immobilization (ii), and nitrite detection (iii) of the assembled hierarchical mesoporous electroenzymatic sensor. Reprinted with permission [129]. Copyright © 2023 Elsevier Ltd. (D) The stepwise fabrication of the chlorophyll-copper (CuCP) modified electrode. Reprinted with permission [132]. Copyright © The Royal Society of Chemistry 2023.

NiR biosensors are designed and fabricated based on natural NiRs and their mimics. Most of these sensors are electrochemical, with wide application potential in environmental monitoring, medical diagnostics, and cellular research [124–126]. By binding the “enzyme” specifically to the electrode surface, researchers leverage its metal active center to catalyze the reduction of nitrite to NO or ammonia. This process is typically detected through cyclic voltammetry (CV) or linear sweep voltammetry (LSV), where changes in current indicate nitrite reduction. Thanks to the enzyme’s high specificity and catalytic efficiency, accurate nitrite detection can be achieved even in complex biological environments. For instance, ccNiR extracted from *Desulfovibrio* is immobilized on a planar electrode (Fig. 13A), achieving rapid response (within 10 s) and a low detection limit of 0.05 μM [127]. Hb immobilized on the Nafion/Hb/AuNRs-WS₂/CILE electrode surface exhibits well-defined redox peaks due to direct electron transfer at the Hb Fe(III)/Fe(II) redox center, resulting in a low nitrite detection limit of 0.33 mM (Fig. 13B) [128]. A drop-cast electrode with NiR (NrfA) from *Shewanella oneidensis* MR-1 has simplified on-site monitoring (Fig. 13C) [129]. To maintain enzyme stability and activity, sensor designs often use physical adsorption, chemical cross-linking, or sol-gel techniques for enzyme immobilization [127,129–131]. Additionally, incorporating nanomaterials (such as carbon nanotubes) can effectively enhance electron transfer efficiency and increase enzyme loading, thereby improving sensor sensitivity and detection limits [132,133].

NiR biosensors, with their high sensitivity, broad detection range, and excellent anti-interference capability, demonstrate important value in biomedical detection. They offer unique advantages, particularly in oral disease screening, real-time monitoring of living cells, and precision diagnostics and treatment. It is known that salivary NO₂^−^ levels are closely associated with oral microbial activity, gingival inflammation, and periodontal disease. For example, excessive NO₂^−^ may indicate the overgrowth of anaerobic oral bacteria (such as periodontal pathogens) or suggest systemic NO metabolism disorders (such as endothelial dysfunction). Manickam et al. have constructed an electrochemical sensor by immobilization of chlorophyll-copper (CuCP) as NiR mimetics on carbon nanotubes (Fig. 13D), effectively detecting nitrite concentrations ranging from 10 μM to 10 mM in standard and human saliva samples [132]. A histamine-coordinated iron phthalocyanine electrochemical microsensor has been developed to sensitively detect NO, with a detection limit as low as 0.03 nM, making it suitable for real-time NO monitoring in living cells [134]. Overall, combining NiR and its mimetics with nanomaterial-based sensor platforms obviously enhances performance in complex environments, making these biosensors powerful tools for nitrite and NO detection, particularly in real-time monitoring and trace analysis.

## Prospects and Challenges

Despite sharing the same fundamental chemistry, NiRs exhibit strikingly different architectures and catalytic idiosyncrasies that both illuminate and complicate our understanding of nitrogen biotransformations. Cu-NiRs, for example, assemble as trimers bristling with 2 distinct copper sites—one “blue” electron-shuttling center and one catalytic copper—yet how electrons navigate between these metals to drive nitrite’s 2-electron reduction to NO (or 6-electron reduction to NH₃ in certain variants) remains elusive. Likewise, iron-based ccNiR and fdNiR diverge in their use of multiheme versus siroheme–[4Fe–4S] cofactors and in their reliance on cytochrome c or ferredoxin as electron donors, underscoring how subunit composition, redox partner specificity, and cellular localization tailor each enzyme to its respiratory or assimilatory niche. Even within a single NiR family, questions persist about how protein conformational changes, proton-relay networks, metal-valence dynamics, and intersubunit contacts finely tune reactivity, selectivity, and intermediate lifetimes.

Looking forward, the field faces both exciting opportunities and formidable challenges. Time-resolved crystallography at x-ray free-electron lasers and room-temperature neutron diffraction promise to capture fleeting intermediates and define protonation states throughout the catalytic cycle. Cryo-electron microscopy (EM) could finally reveal how subunit interactions modulate activity in trimeric Cu-NiRs, while advanced spectroscopies coupled with computational modeling may map electron–proton coupling pathways in exquisite detail. Clinically, harnessing or modulating endogenous NiR activity offers a tantalizing avenue for disease intervention: engineered or small-molecule-activated Cu-NiRs might serve as on-demand NO donors to treat hypertension or ischemia, whereas selective inhibition of bacterial NiRs could attenuate pathogen-derived ammonia production and reduce virulence. As we bridge mechanistic insights with translational goals, a deeper grasp of NiR diversity and regulation will be pivotal to unlocking both their environmental significance and their untapped therapeutic potential.

Artificial NiR mimics hold promise for catalytic NO-based therapies and nitrite biosensing but face critical hurdles in replicating enzyme-level activity and selectivity under physiological conditions. They must balance biomimetic complexity with synthetic tractability: replicating distorted coordination geometries and mixed-valence Cu/Fe clusters remains elusive using traditional coordination chemistry, leading to low yields and batch variability. Under physiological or harsh conditions, many nanozymes suffer metal leaching or framework collapse, compromising long-term activity. Moreover, the absence of dynamic substrate-binding pockets in synthetic catalysts weakens nitrite affinity, causing off-pathway reactions such as over-reduction to NH₃ or N₂O. In biological milieu, oxygen and reactive oxygen species (ROS) competitively intercept electrons, and blood proteins or thiols deactivate catalysts, undermining both therapeutic NO release and sensing accuracy.

To address these issues, supramolecular assemblies—MOFs and covalent organic frameworks (COFs)—enable precise preorganization of ligands and metal nodes, faithfully mimicking enzyme pockets and stabilizing active centers against leaching. Hybrid materials, such as embedding Cu or Fe complexes in polymeric or carbon matrices, bolster structural integrity and resist collapse, while artificial intelligence-assisted design accelerates discovery of optimal ligand–metal combinations, reducing trial-and-error cycles and enhancing synthetic yields. For example, a comprehensive nanozyme database has been created by synthesizing insights from extensive literature, and cutting-edge methodologies—ranging from quantum-mechanical reaction-pathway analyses to machine-learning-driven optimization—have been deployed to identify and refine the most effective catalytic routes, forging a new paradigm in rational nanozyme design.[135]. Introducing substrate-selective binding via molecular imprinting or hydrophilic/hydrophobic microenvironments (e.g., porous MOFs) recreates enzyme-like specificity, minimizing side reactions. Synergistic catalytic site architectures—incorporating bimetallic centers or redox mediators like quinones—streamline proton-coupled electron transfer, elevating selectivity toward NO versus unwanted by-products. Finally, stimuli-responsive systems (pH-, ROS-, or enzyme-activated) ensure NO generation only in disease-relevant microenvironments, while surface functionalization (PEGylation and cell-membrane coatings) and targeted nanocarriers (liposomes, dendrimers, and mesoporous silica) enhance biocompatibility, evade immune clearance, and exploit the EPR effect for tumor or infection site accumulation.

In the future, integrating these strategies into “intelligent” nanozyme platforms that dynamically adjust catalytic output in response to real-time physiological cues will be key for translating NiR mimics into safe and effective NO therapeutics and reliable nitrite biosensors. Continuous development in ultrafast structural characterization (x-ray free-electron laser and cryo-EM), advanced spectroscopy, and computational modeling will underpin this progress by illuminating active-site dynamics and guiding the rational design of next-generation NiR mimics.
